# The omics approach to bee nutritional landscape

**DOI:** 10.1007/s11306-019-1590-6

**Published:** 2019-09-20

**Authors:** Priyadarshini Chakrabarti, Jeffery T. Morré, Hannah M. Lucas, Claudia S. Maier, Ramesh R. Sagili

**Affiliations:** 1 Department of Horticulture, Oregon State University, Corvallis, OR 97331 USA; 2 Department of Chemistry, Oregon State University, Corvallis, OR 97331 USA

**Keywords:** Bee nutrition, Crop pollens, Metabolomics, Phytosterols, Vegetable oils, Protein supplements

## Abstract

**Background:**

Significant annual honey bee colony losses have been reported in the USA and across the world over the past years. Malnutrition is one among several causative factors for such declines. Optimal nutrition serves as the first line of defense against multiple stressors such as parasites/pathogens and pesticides. Given the importance of nutrition, it is imperative to understand bee nutrition holistically, identifying dietary sources that may fulfill bee nutritional needs. Pollen is the primary source of protein for bees and is critical for brood rearing and colony growth. Currently, there is significant gap in knowledge regarding the chemical and nutritional composition of pollen.

**Methods:**

Targeted sterol analysis and untargeted metabolomics were conducted on five commercially available crop pollens, three bee-collected crop pollens, three vegetable oils (often added to artificial protein supplements by beekeepers), and one commonly used artificial protein supplement.

**Results:**

This study reports key phytosterols and metabolites present across a spectrum of bee diets, including some of the major bee-pollinated crop pollens in the western United States. Significant differences were observed in sterol concentrations among the dietary sources tested. Among all quantified sterols, the highest concentrations were observed for 24-methylenecholesterol and further, pollen samples exhibited the highest 24-methylenecholesterol among all diet sources that were tested. Also, 236 metabolites were identified across all dietary sources examined.

**Conclusion:**

Information gleaned from this study is crucial in understanding the nutritional landscape available to all bee pollinators and may further assist in future efforts to develop comprehensive database of nutrients and metabolites present in all bee diets.

**Electronic supplementary material:**

The online version of this article (10.1007/s11306-019-1590-6) contains supplementary material, which is available to authorized users.

## Introduction

High annual losses of honey bee colonies (*Apis mellifera*) and native bee population declines have been reported, both in the United States and globally (Frazier et al. 2008; Cameron et al. 2011; Kulhanek et al. 2017), with over 40% managed honey bee colony losses in the United States alone in 2017–2018 (The Bee Informed Partnership 2019). These losses have been attributed to multiple stressors; but among those commonly cited as major causes is poor nutrition. Honey bee colonies with access to adequate amounts of high quality pollen have lower pathogen loads, have higher brood area, overwinter more successfully and are less susceptible to the gut parasite *Nosema ceranae* than those receiving scanty or poor quality nutrition (Matilla and Otis 2006; Eischen and Graham 2008; Girard et al. 2012; Di Pasquale et al. 2013; Jack et al. 2016; Glavinic et al. 2017). With increasing pressure to halt and reverse pollinator declines, the consequences of malnutrition on bee health must be thoroughly examined (Goulson et al. 2015; Perry et al. 2015; Steinhauer et al. 2018).

Pollen is a critical component of vascular plant reproduction; it is equally important to bee pollinators because it is their primary source of proteins, lipids, vitamins, minerals and vital phytochemicals (Matilla and Otis 2006; Brodschneider and Crailsheim 2010; Scofield and Mattila 2015; Vaudo et al. 2015; Arathi et al. 2018). While foraging for pollen, bees provide the critical ecosystem service of pollination; in fact, they are the most efficient of all insect pollinators (Free 1970). In spite of its importance to bees and plants, there exists a significant gap in knowledge regarding chemical and nutritional constituents of pollen (Arathi et al. 2018).

Commercial honey bee colonies are intensely managed and manipulated. Hives are repeatedly moved between blooming crops to meet pollination needs (Topitzhofer et al. 2019). The logistics of these migrations impose additional stress on the honey bees (vanEngelsdorp et al. 2012; Tarpy et al. 2013; Simone-Finstrom et al. 2016). Commercial management of honey bee colonies also involves supplementary feeding during pollen or nectar dearth (Standifer et al. 1977; Honey Bee Health Coalition report 2017). Beekeepers often provide commercially available protein supplements to their colonies or feed them their own protein patty formulations mixed with vegetable oils (Sammataro and Avitabile 1998). Native bee pollinators are also dependent on pollen for their sustenance, but unfortunately do not have supplementary feeding support that is received by domesticated honey bees. Therefore, understanding the effects of nutrition on bee health must include not only an examination of the nutritional composition of floral pollen but also a similar evaluation of the protein supplements and vegetable oils fed to honey bees.

In addition to plant secondary metabolites, amino acids and vital phytochemicals, pollens provide phytosterols that are critical for molting hormone production, cell membrane stability and other vital functions in bee physiology (Behmer and Nes 2003; Carvalho et al. 2010; Arathi et al. 2018). It has previously been reported that 24-methylenecholesterol is vital for honey bees (Herbert et al. 1980; Svoboda et al. 1980; Feldlaufer 1986). Similarly, 24-methylenecholesterol, β-sitosterol and δ5-avenasterol appear to be important to bumble bee micro-colonies (Vanderplanck et al. 2014). Our knowledge regarding sterol requirements in bee species is limited; nevertheless, it remains important to understand the sterol composition of all potential dietary sources (pollen and protein supplements) of these macromolecules. Such understanding may lead to practical methods for improving bee health through better nutrition. For example, understanding the sterol profile of pollens available to bees may help in selection of appropriate plant species to be planted as supplemental forage near bee-pollinated crops that do not themselves provide adequate nutrition. It could also identify better quality (for example high phytosterol content) pollens that could be collected for use as substitutes by beekeepers during pollen dearth.

The main goal of this study was to better understand the wider spectrum of nutrients available to all bee species via different dietary sources, thereby creating a foundation on which a more holistic understanding of bee nutrition can be built. Here we provide a comparative description of the metabolites found in bee dietary sources (pollens, vegetable oils and protein supplements). We also report the relative quantities of ten different phytosterols and cholesterol among the nutritional sources tested. To our knowledge, this is the first study to analyze phytosterols and metabolites across a wide range of bee dietary sources—pollens from crops dependent on bee-pollination (commercially available pollens and bee-collected pollens), a commercial honey bee protein diet and three vegetable oils commonly added to honey bee protein supplements. Further, the methods established in this study could be extended to examine phytosterols and metabolites in other bee diets (pollens or protein supplements) in the future.

## Materials and methods

### Sample collections

Commercial pollen samples were procured from Firman Pollen (monospecific pure pollen; Firman Pollen Co Inc, Yakima, USA). The following crop pollens were purchased in 2018: plum, almond, apricot, apple and cherry. These crops were chosen because they are highly dependent on bees for pollination (Klein et al. 2007; Giannini et al. 2015). For obtaining bee-collected pollen (referred to as corbicular pollen for the remainder of this paper), pollen traps were installed at the entrances of three honey bee colonies, for 24 h during peak bloom, in each of the following pollinator-dependent crops in 2018: blueberry and pear in Oregon and almond in California. The following vegetable oils were commercially purchased in 2018 based on beekeepers’ practices of protein patty formulations: borage (Naissance Virgin Borage, Neath, UK), canola (Crisco, Orrville, USA) and soybean (Crisco, Orrville, USA). One commonly used commercial bee diet (protein supplement) was also purchased in 2018. Three biological replicates were tested for each type of dietary sample (commercial and corbicular pollen, commercial diet and vegetable oils). For corbicular pollen samples, one replicate per colony was tested for each crop. A quick summary of the sample types and number of replicates are provided in Table 1. For each sample type, mean values were calculated from the sterol analysis data of all three replicates. All the pollen samples were transported on dry ice and were immediately stored at − 20 °C (Amana Deep Freezer, Benton Harbor, USA) until further analysis.

**Table 1:** Sampling methodology detailing the dietary sample types and replicate numbers for each

Dietary sample type	Number of replicates
Commercial pollen	
Plum	3
Almond	3
Apricot	3
Apple	3
Cherry	3
Bee-collected (corbicular) pollen	
Almond	3
Pear	3
Blueberry	3
Vegetable oils	
Borage	3
Canola	3
Soybean	3
Commercial diet	3

### Targeted analysis of sterols

#### Sample preparation

Twenty milligrams of each sample replicate was homogenized in one milliliter of 25:10:65 v/v/v solution of methylene chloride, isopropanol and methanol (Thermo Fisher Scientific, Grand Island, USA). Samples were homogenized in a Precellys 24 tissue homogenizer (Bertin Instruments, Rockville, USA) at 6000 rpm for three cycles of 30 s cycle^−1^. Next, the homogenized samples were incubated for 1 h at − 20 °C and then centrifuged in an Eppendorf centrifuge (5430R, Eppendorf, USA) at 13,000 rpm for 10 min at 4 °C. The supernatant was collected in HPLC sample vials (MicroSolv Technology Corporation, Leland, USA) and stored in − 20 °C until analysis. The following eight sterols were purchased from Avanti (Avanti Polar Lipids, Alabaster, USA)—cholesterol, delta-5-avenasterol, desmosterol, 24-methylenecholesterol, sitostanol, campesterol, campestanol and brassicasterol. The other three sterols—ergosterol, β-sitosterol and stigmasterol—were purchased from TCI America (TCI America, Portland, USA). Calibration curves for each sterol were prepared at concentrations of 1 µM, 5 µM, 10 µM, 50 µM and 75 µM in acetonitrile (Thermo Fisher Scientific, Grand Island, USA). The following deuterated sterols were also purchased from Avanti (Avanti Polar Lipids, Alabaster, USA)—desmosterol-d6, sitosterol-d7 and cholesterol-d7. All three deuterated standards were added to each sterol standard mix as a final concentration of 10 µM to check the efficacy of our separation process.

#### Liquid chromatography multiple reaction monitoring (LC-MRM) analysis of sterols

Sterol profiles were analyzed using Liquid Chromatography-Atmospheric Pressure Chemical Ionization-Multiple Reaction Monitoring (LC-APCI-MRM) methods at the Oregon State University Mass Spectrometry Center (OSUMSC). The MRM transitions monitored were based on protocols by Agilent Technologies (Fu and Joseph 2012). Eleven sterols and three deuterated standards were identified and quantified based on their MRM transitions and retention times (Table 2). The targeted sterols were separated using an isocratic gradient using an Agilent Poroshell 120 EC-C18 column (3 mm × 100 mm, 2.7 µm; Agilent, Santa Clara, USA). The mobile phase was 80% acetonitrile:20% methanol:0.1% formic acid (Thermo Fisher Scientific, Grand Island, USA) with a flow rate of 0.6 mL/min. Three microliters of each sample were injected using a Shimadzu Prominence HPLC (Shimadzu, Columbia, USA) coupled to an Applied Biosystem 4000 Qtrap mass spectrometer (AB SCIEX, Foster City, USA) operated in the APCI-MRM positive ionization mode. The APCI source temperature was set at 350 °C, declustering potential was 71 V, entrance potential was 10 V, collision cell exit potential was 9 V, nebulizing gas 1 (GS1) was 30 L/min, curtain gas was set at 20 and nebulizer current was 3.0 µA. Precursor ion [M + H−H_2_O]^+^ intensity in the APCI mode was optimized for the loss of H_2_O from the protonated molecular ion [M + H]^+^ based on previous studies (McDonald et al. 2012). Sterol data was analyzed using Analyst™ TF 1.7.1 (AB SCIEX, Foster City, USA) and MultiQuant™ 3.0.2 (AB SCIEX, Foster City, USA). Mean values for sterol concentrations are reported in ppm with standard errors for means.

**Table 2:** Multiple reaction monitoring (MRM) transitions and retention times for the sterols analyzed in the present study

Sterol	Precursor ion [M + H–H_2_O]^+^ (m/z)	Product ions monitored (m/z)	Collision energy (V) (1st transition; 2nd transition)	Retention times (min)
Cholesterol	369.4	161.1; 95.1	30; 50	4.95
Cholesterol-d7	376.4	161.1; 95.1	30; 50	4.91
Campesterol	383.4	161.1; 95.1	30; 50	5.62
Desmosterol	367.4	161.1; 95.1	30; 50	3.59
Desmosterol-d6	373.4	161.1; 95.1	30; 50	3.56
Stigmasterol	395.4	83.1; 81.1	30; 30	4.77
β-Sitosterol	397.4	161.1; 135.1	30; 30	6.45
Sitosterol-d7	404.4	161.1; 135.1	30; 30	6.42
Campestanol	385.4	189.3; 135.2	30; 30	6.71
Ergosterol	379.4	69.1; 189.3	40; 60	3.87
24-Methylenecholesterol	381.4	105.1; 161.1	60; 50	3.98
Sitostanol	399.4	135.1; 95.1	30; 40	7.75
Δ5-Avenasterol	395.5	69.0; 93.3; 147.2	60; 60; 40	4.82
Brassicasterol	381.5	69.0; 95.1; 147.2	60; 40; 40	4.79

[M + H−H_2_O]^+^ indicates the protonated molecular ion after loss of water

### Analysis of the metabolome (untargeted metabolomics)

#### Sample preparation

For each sample type, 50 mg of each replicate were homogenized in 0.5 mL solution of methanol and water (80:20, v/v; Thermo Fisher Scientific, Grand Island, USA), which extracted both polar and nonpolar metabolites. The samples were homogenized in a Precellys 24 tissue homogenizer (Bertin Instruments, Rockville, USA) at 6000 rpm for three cycles at 30 s/cycle. The homogenized samples were incubated for 1 h at − 20 °C, centrifuged at 14,000 rpm at 4 °C for 5 min and 400 µL of the supernatant was collected and evaporated to dryness in a speed vacuum concentrator (CentriVap, Labconco, Kansas City, USA). The dry extracts were reconstituted in 200 µL of 1:1 v/v acetonitrile and water, vortexed for 30 s and centrifuged at 4 °C for 5 min at 13,000 rpm. The supernatants were transferred to HPLC vials (MicroSolv Technology Corporation, Leland, USA) and stored in − 80° C (VWR, USA) until analysis.

#### Mass spectrometry of metabolites

Metabolomics experiments were performed based on previous studies (Kirkwood et al. 2016) using a Nexera LC30 UPLC (Shimadzu, Columbia, USA) coupled to a quadrupole-time-of-flight mass spectrometer (TripleTOF 5600, AB SCIEX, Foster City, USA) at the OSUMSC. Data was acquired in the Information Dependent Acquisition (IDA) mode. Samples were analyzed in the positive and negative ionization modes. External mass calibration was conducted automatically every 2 h. Metabolites were separated using an Inertsil Phenyl-3 stationary phase column (150 mm × 4.6 mm, 5 µm; GL Sciences, Washington D.C., USA). The column temperature was held at 50° C. Three microliters of each sample were injected. Metabolite elution was achieved using a gradient as previously described (Kirkwood et al. 2016): solvent A was 100% water containing 0.1% formic acid and solvent B was 100% methanol containing 0.1% formic acid. Flow rate was 0.4 mL/min. Metabolites were tentatively identified using an in-house library based on IROA’s Mass Spectrometry Metabolite Library of Standards (IROA Technologies, Boston, USA) using accurate mass, fragmentation pattern, isotope distribution and retention time. Further putative metabolite assignments were conducted using Progenesis QI software (Nonlinear Dynamics, Durham, USA) with METLIN™ MS/MS spectral library plugin. The data was quantitated using Analyst™ TF 1.7.1 (AB SCIEX, Foster City, USA) and MultiQuant™ 3.0.2 (AB SCIEX, Foster City, USA) and the data was further analyzed using PeakView™ 2.2.0 (AB SCIEX, Foster City, USA) and MarkerView™ 1.2.1.1 software (AB SCIEX, Foster City, USA).

### Statistical analysis

All statistical analyses for sterol data were performed using R version 3.3.3 and Graph Pad Prism v8.0.1. Shapiro–Wilk test was conducted to test for normality. The data was log transformed, if found to be not normal. One-way ANOVA was performed for each sterol, with multiple comparisons by Tukey’s Post Hoc tests. For metabolomics, all data were log transformed and the transformed data were analyzed using MetaboAnalyst v4.0 to generate heat maps, dendrogram clusters, correlation matrix and significance analysis of microarrays plot (SAM plot). Principle component analysis (PCA) plots were generated in MarkerView™ Software v1.2.1.1 (AB SCIEX, Foster City, USA). Means are presented as ± standard errors for means.

## Results

### Targeted analysis of sterols

**Fig. 1:**
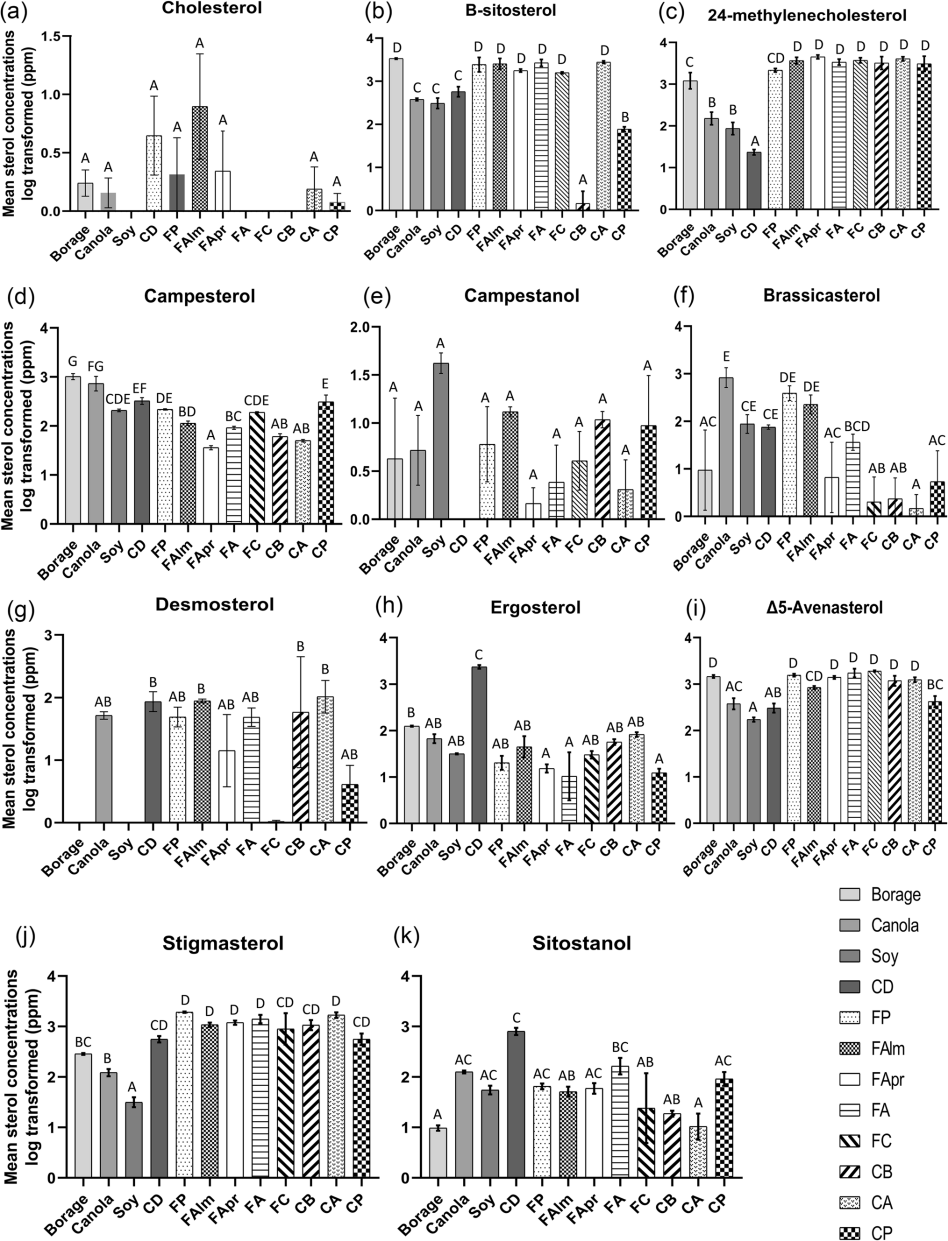
Mean concentrations (ppm) of all sterols quantified in all dietary samples. *CA* corbicular almond pollen, *CB* corbicular blueberry pollen, *CD* commercial diet, *CP* corbicular pear pollen, *FA* firman apple pollen, *FAlm* firman almond pollen, *FApr* firman apricot pollen, *FC* firman cherry pollen, *FP* firman plum pollen, *Soy* soybean oil, *Borage* borage oil and *Canola* canola oil. Results from one-way ANOVA and Tukey’s Post Hoc tests are indicated in the graphs, where, similar alphabets indicate no significant differences between the sample types for a particular sterol tested. Means are presented as mean ± SEM

Significant differences were observed in sterol concentrations among the dietary sample types (Fig. 1) for 24-methylenecholesterol (F_(11,24)_ = 134.3, p < 0.001), brassicasterol (F_(11,24)_ = 13.45, p < 0.001), β-sitosterol (F_(11,24)_ = 203.6, p < 0.001), campesterol (F_(11,24)_ = 39.84, p < 0.001), Δ5-avenasterol (F_(11,24)_ = 21.33, p < 0.001), desmosterol (F_(11,24)_ = 5.988, p < 0.001), ergosterol (F_(11,24)_ = 12.52, p < 0.001), sitostanol (F_(11,24)_ = 5.507, p < 0.001) and stigmasterol (F_(11,24)_ = 21.77, p < 0.001). No significant differences were observed for cholesterol (F_(11,24)_ = 1.585, p = 0.167) and campestanol (F_(11,24)_ = 1.844, p = 0.102). There were no significant differences in sterol concentrations between commercial and corbicular almond pollens, except for brassicasterol (Fig. 1). Table 3 presents the retention times and the mean concentrations of sterols analyzed. A total ion chromatogram, indicating the identified sterols for a representative sterol standard mix (75 µM), is presented in supplementary Fig. 1. In particular, 24-methylenecholesterol was present in high concentrations in pollen samples (commercial and corbicular) with values ranging between 216.34 ± 12.38 ppm (commercial plum pollen) to 407.91 ± 26.05 ppm (corbicular almond pollen). The concentrations of 24-methylenecholesterol also differed among the vegetable oils with 15.79 ± 3.35 ppm, 8.99 ± 1.57 ppm and 128.89 ± 30.02 ppm detected in canola, soybean and borage oil, respectively. The commercial diet had a low concentration of 24-methylenecholesterol (2.36 ± 0.19 ppm). In pollen samples, β-sitosterol and Δ5-avenasterol concentrations were the next highest sterol concentrations after 24-methylenecholesterol. In vegetable oil samples—after 24-methylenecholesterol—campesterol, β-sitosterol and Δ5-avenasterol were found in relatively higher concentrations. For the commercial diet, the following sterols were present in high concentrations: ergosterol (239.29 ± 20.61 ppm), β-sitosterol (58.83 ± 9.54 ppm), stigmasterol (57.32 ± 8.68 ppm), sitostanol (82.53 ± 13.55 ppm) and campesterol (33.24 ± 5.59 ppm). Figure 1 presents the mean concentrations of eleven sterols detected across the dietary samples. The detailed results from Tukey’s Post Hoc comparisons have been provided in supplementary information (supplementary_dataset_1).

**Table 3:** Eleven sterols quantified from the various honey bee diet sources are shown as mean values of concentrations in ppm ± standard errors of means

Sterols	Choles	βsito	24MC	Cpsterol	Cpstanol	Brassica	Desmo	Ergo	Δ5	Stigma	Sito
Retention time (min)	4.95	6.45	3.98	5.62	6.71	4.79	3.59	3.87	4.82	4.77	7.75
Commercial pollen (ppm)											
Plum	Trace	257.30 ± 61.72	216.34 ± 12.38	21.77 ± 0.16	0.99 ± 0.51	40.86 ± 8.74	5.54 ± 1.74	2.28 ± 0.76	157.36 ± 10.03	192.70 ± 5.70	6.63 ± 0.81
Almond	Trace	261.81 ± 45.27	372.64 ± 39.48	11.51 ± 1.26	0.63 ± 0.17	24.33 ± 6.64	8.93 ± 0.62	5.59 ± 2.02	85.05 ± 6.61	109.68 ± 11.03	5.37 ± 1.06
Apricot	Trace	178.06 ± 8.16	454.14 ± 28.98	2.64 ± 0.36	Trace	1.26 ± 0.79	3.61 ± 1.82	1.59 ± 0.32	141.21 ± 10.75	120.39 ± 11.03	6.29 ± 1.44
Apple	ND	271.52 ± 29.92	341.26 ± 31.46	9.24 ± 0.65	Trace	3.83 ± 0.78	5.41 ± 1.55	2.39 ± 1.39	181.63 ± 35.42	146.00 ± 28.49	18.68 ± 6.16
Cherry	ND	157.65 ± 4.49	377.01 ± 32.71	19.05 ± 0.54	0.55 ± 0.28	Trace	Trace	3.13 ± 0.59	192.13 ± 7.26	129.50 ± 54.28	7.95 ± 4.04
Corbicular pollen (ppm)											
Blueberry	ND	Trace	334.27 ± 60.82	6.22 ± 0.82	1.13 ± 0.23	Trace	31.22 ± 17.41	5.81 ± 0.81	126.18 ± 32.18	112.39 ± 24.38	1.91 ± 0.27
Pear	ND	7.78 ± 0.60	325.78 ± 67.78	34.63 ± 11.97	2.46 ± 1.81	0.88 ± 0.50	0.56 ± 0.28	1.29 ± 0.29	45.35 ± 11.61	59.77 ± 15.03	10.11 ± 3.08
Almond	ND	278.52 ± 10.93	407.91 ± 26.05	5.03 ± 0.29	Trace	Trace	14.29 ± 7.18	8.38 ± 0.93	126.65 ± 15.56	171.19 ± 22.15	1.37 ± 0.57
Vegetable oil (ppm)											
Borage	Trace	33.68 ± 6.14	128.89 ± 30.02	103.53 ± 13.44	2.59 ± 2.58	1.93 ± 0.97	ND	12.45 ± 0.41	147.41 ± 10.42	28.76 ± 1.18	0.98 ± 0.11
Canola	Trace	37.98 ± 1.49	15.79 ± 3.35	82.39 ± 28.27	0.81 ± 0.43	89.60 ± 24.10	5.30 ± 0.68	7.05 ± 1.40	41.03 ± 11.88	12.52 ± 1.89	12.66 ± 0.83
Soybean	ND	31.87 ± 5.39	8.99 ± 1.57	20.94 ± 1.39	4.39 ± 0.95	9.39 ± 2.24	ND	3.16 ± 0.10	17.43 ± 1.91	3.29 ± 0.65	5.75 ± 1.16
Commercial diet (ppm)											
Pollen supplement	0.67 ± 0.19	58.83 ± 9.54	2.36 ± 0.19	33.24 ± 5.59	ND	7.60 ± 0.44	9.82 ± 3.34	239.29 ± 20.61	32.39 ± 7.77	57.32 ± 8.68	82.53 ± 13.55

ND indicates not detected (0 ppm). Trace indicates concentrations less than 0.5 ppm. The statistical details are provided in supplementary dataset 1

*Choles* cholesterol, *βsito* β-sitosterol, *24MC* 24-methylenecholesterol, *Cpsterol* campesterol, *Cpstanol* campestanol, *Brassica* brassicasterol, *Desmo* desmosterol, *Ergo* ergosterol, *Δ5* Δ5-avenasterol, *Stigma* stigmasterol, *Sito* sitostanol

### Analysis of the bee diet metabolomes

**Fig. 2:**
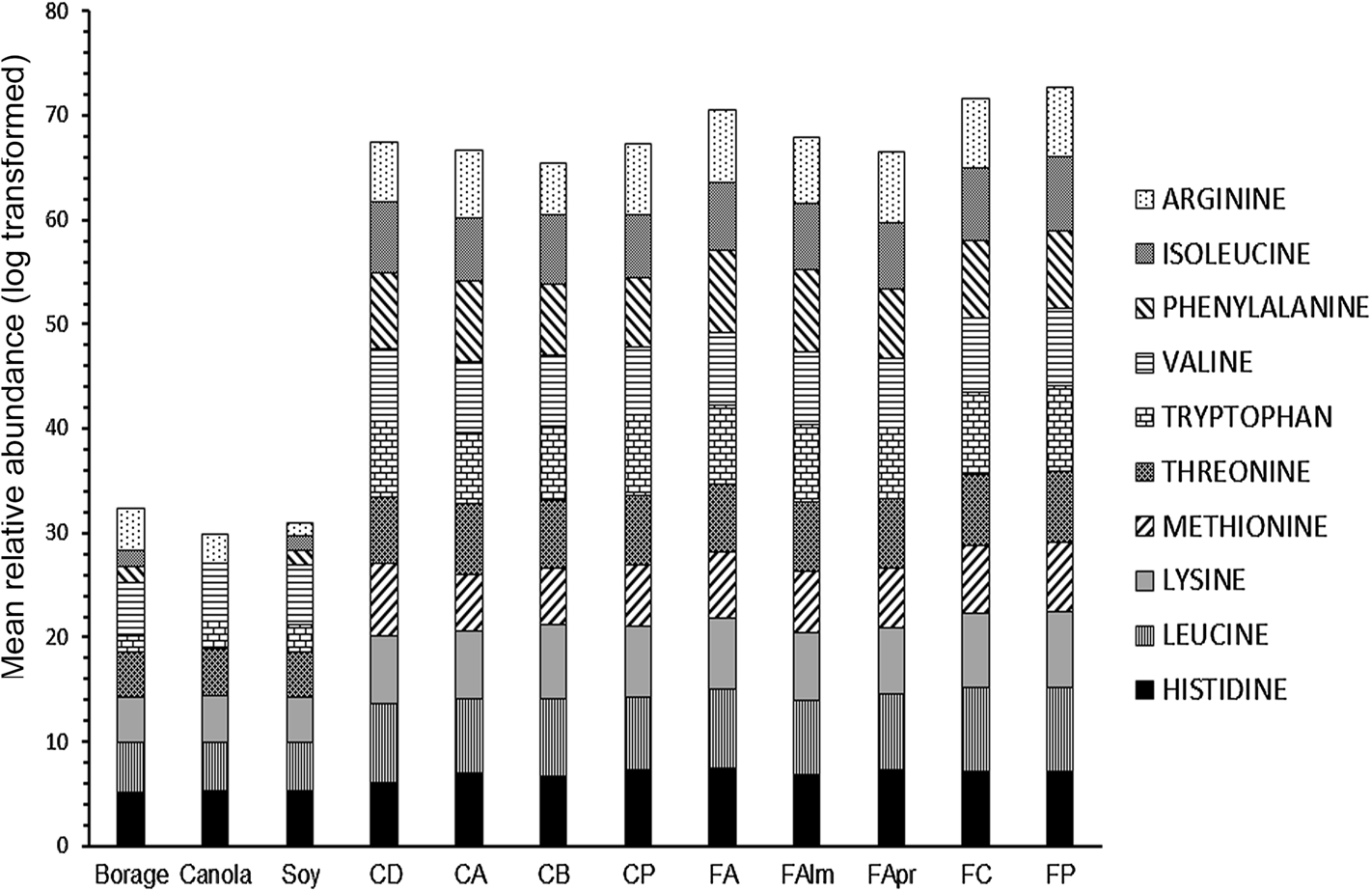
Log transformed mean relative abundance of ten essential amino acids in all dietary samples. *CA* corbicular almond pollen, *CB* corbicular blueberry pollen, *CD* commercial diet, *CP* corbicular pear pollen, *FA* firman apple pollen, *FAlm* firman almond pollen, *FApr* firman apricot pollen, *FC* firman cherry pollen, *FP* firman plum pollen, *Soy* soybean oil, *Borage* borage oil and *Canola* canola oil

A total of 236 metabolites were tentatively assigned across all the dietary sources, using data from both the positive (131) and the negative (105) ionization modes. Supplementary Table 1 presents the detailed list of tentatively assigned metabolites across the various samples. The heat maps (supplementary Figs. 2 and 3) and dendrograms (supplementary Figs. 4 and 5) generated using the relative abundance values of the metabolites, for both ionization modes, further corroborate the results obtained in PCA plots (supplementary Fig. 6). The vegetable oil samples were distinctly different from the pollen samples (both commercially purchased and corbicular pollens) as well as the commercial diet. The commercial diet was found to be an intermediate between pollens and vegetable oils, in terms of its metabolite contents. As evident from the heat maps, a relative low abundance of tentatively assigned metabolites was found in the vegetable oil samples. The correlation matrix is shown in supplementary Fig. 7 and the SAM plot is presented in supplementary Fig. 8. The correlation index, obtained from the correlation matrix, demonstrates that pollen samples exhibit the strongest correlations between each other, irrespective of the source (commercial or corbicular) or type of plant (CI 0.7–1). The SAM plot for multiple testing reveals that 199 metabolites were found in significantly different concentrations across various dietary samples. The relative abundances of an important phenolic acid—p-coumaric acid—was significantly different across the sample types (one-way ANOVA, F_(11,24)_ = 225.8, p < 0.001) and was lower in vegetable oils and commercial diet samples than in pollens (commercial and corbicular). The flavonol kaempferol was also significantly different among the samples (one-way ANOVA, F_(11,24)_ = 22.30, p < 0.001). It was present in relatively low abundance in canola and borage oil and was not detected in soybean oil samples. Supplementary Fig. 9 depicts a representative total ion chromatograph, highlighting some major groups of metabolites identified across different retention times. Figure 2 presents the relative abundances of 10 essential amino acids from all dietary samples. The essential amino acid composition of the commercial diet was similar to the pollen samples. The vegetable oils contained very few amino acids. The percentages of crude proteins of these dietary sample types, sourced from existing literature, are provided in Table 4.

**Table 4:** Crude protein estimates of the dietary samples tested

Dietary sample type	Crude protein (%)	Source
Commercial pollen		
Plum^a^	24.43	Forcone et al. (2011)
Almond^b^	30.5	Topitzhofer (2014)
Apricot^a^	24.43	Forcone et al. (2011)
Apple	25.12	Pernal and Currie (2000)
Cherry^a^	24.43	Forcone et al. (2011)
Bee-collected (corbicular) pollen		
Almond	30.5	Topitzhofer (2014)
Pear	26	Somerville (2001)
Blueberry	14	Somerville (2001)
Vegetable oils		
Borage	0	Product label information
Canola	0	Product label information
Soybean	0	Product label information
Commercial diet	18	Product label information

^a^Crude pollen protein estimates are based on Rosaceae family pollen crude proteins reported

^b^Commercial almond crude pollen protein estimates are based on almond pollen existing information from available literature

## Discussion

Pollen—primarily comprised of lipids (including phytosterols), proteins, vitamins and plant secondary metabolites—is an integral part of bees’ natural diets (Roulston et al. 2000; Arathi et al. 2018). Our study reports novel findings regarding composition of sterols and metabolites in select crop pollens, a commercially available protein supplement and vegetable oils that are commonly mixed in these protein supplements by beekeepers. The diet components varied in their phytosterol and metabolite compositions. Understanding the nutrient composition of protein sources for bees (pollen and protein supplements) is crucial, as protein is vital for development of larvae in all bees. Pollen generalist bees may rectify nutrient imbalance and mitigate potential harmful effects of secondary metabolites by providing mixed pollen (diverse pollen) to their larvae (Eckhardt et al. 2014).

Several studies have reported beneficial effects of the sterols and metabolites that were detected in our study (for example 24-methylenecholesterol, p-coumaric acid, kaempferol, essential amino acids etc.) on bee gut microbiota and overall bee health (Herbert et al. 1980; Olofsson and Vásquez 2008; Vásquez and Olofsson 2009; Liao et al. 2017; Bernklau et al. 2019). Hence, it is imperative to understand the actual nutrient composition of these diets that are critical for honey bee physiology, health and immune system.

Our study did not find any significant differences in any of the sterol concentrations, except brassicasterol, between commercially available (hand collected) pollen and honey bee collected (corbicular) pollen for almond. This suggests that pollen collection method did not largely affect the sterol concentrations for this particular crop pollen. The only difference in brassicasterol may be because bee-collected almond pollen pellet is primarily constituted of almond pollen (Topitzhofer et al. 2019), but commercial pollen purchased for this study was of pure strength. Further, our findings suggest that borage oil has relatively high concentrations of 24-methylenecholesterol when compared to other commonly supplemented oils, and therefore may serve as a valuable additive to honey bee artificial protein diets, even though it lacks amino acids and has relatively low quantities of important metabolites. Our findings corroborate the results of another study (Reina et al. 1999) that found a high percentage of 24-methylenecholesterol in borage oil. The other two vegetable oils tested are not only insufficient protein sources, but also lack in critically required phytosterols. The limited abundance of amino acids in vegetable oils may be a result of industrial processing. Therefore, one may assume that these vegetable oils may not be adequate for honey bee dietary supplementations, with respect to required proteins and sterols. For both pollen groups (commercial and corbicular), except blueberry pollen, all pollen sources have high crude proteins reported. The commercial diet reports higher crude proteins than vegetable oils and blueberry pollens, but still lacks in comparison to other plant pollens tested in the present study.

Some studies have also demonstrated the importance of amino acid and sterol composition of pollen in bumble bees. Bumble bee micro-colonies fed pollens with higher concentrations of 24-methylenecholesterol and total amino acids, produced bigger larvae and rapidly developed into strong colonies (Vanderplanck et al. 2014; Moerman et al. 2016). The corbicular pollen collected from honey bees is commonly used to supplement honey bee colonies during pollen dearth and rear commercially available bumble bees and solitary bees (Hoover and Ovinge 2018; Arathi et al. 2018). Selecting such pollens, based on an understanding of their nutritional profiles, could therefore improve the commercial scale production of these important bee pollinators.

Our study is a step towards developing a comprehensive database of nutrients and metabolites present in diets (pollen and artificial diets) of bees. Future research should focus on deciphering the function/role of these nutrients and metabolites in the physiology and survival of bees. Such information could be used to formulate a balanced/optimal diet for managed honey bees and also potentially assist in planning and planting suitable forage for both managed and native bees.

## Electronic supplementary material

Below is the link to the electronic supplementary material.

Supplementary material 1 (TIFF 721 kb)

Supplementary material 2 (TIFF 2659 kb)

Supplementary material 3 (TIFF 1845 kb)

Supplementary material 4 (TIFF 1321 kb)

Supplementary material 5 (TIFF 1456 kb)

Supplementary material 6 (TIFF 1774 kb)

Supplementary material 7 (TIFF 2450 kb)

Supplementary material 8 (TIFF 524 kb)

Supplementary material 9 (TIFF 15627 kb)

Supplementary material 10 (DOCX 40 kb)

Supplementary material 11 (PDF 117 kb)

## Data Availability

All data generated or analyzed during this study are included in this published article (and its supplementary information files). Any other information is available from the corresponding author on reasonable request.
