# Supplementary figures and images for: The omics approach to bee nutritional landscape
Source: Metabolomics. 2019 Sep 20;15(10):127. doi: 10.1007/s11306-019-1590-6 (PMC6753177; doi:10.1007/s11306-019-1590-6)

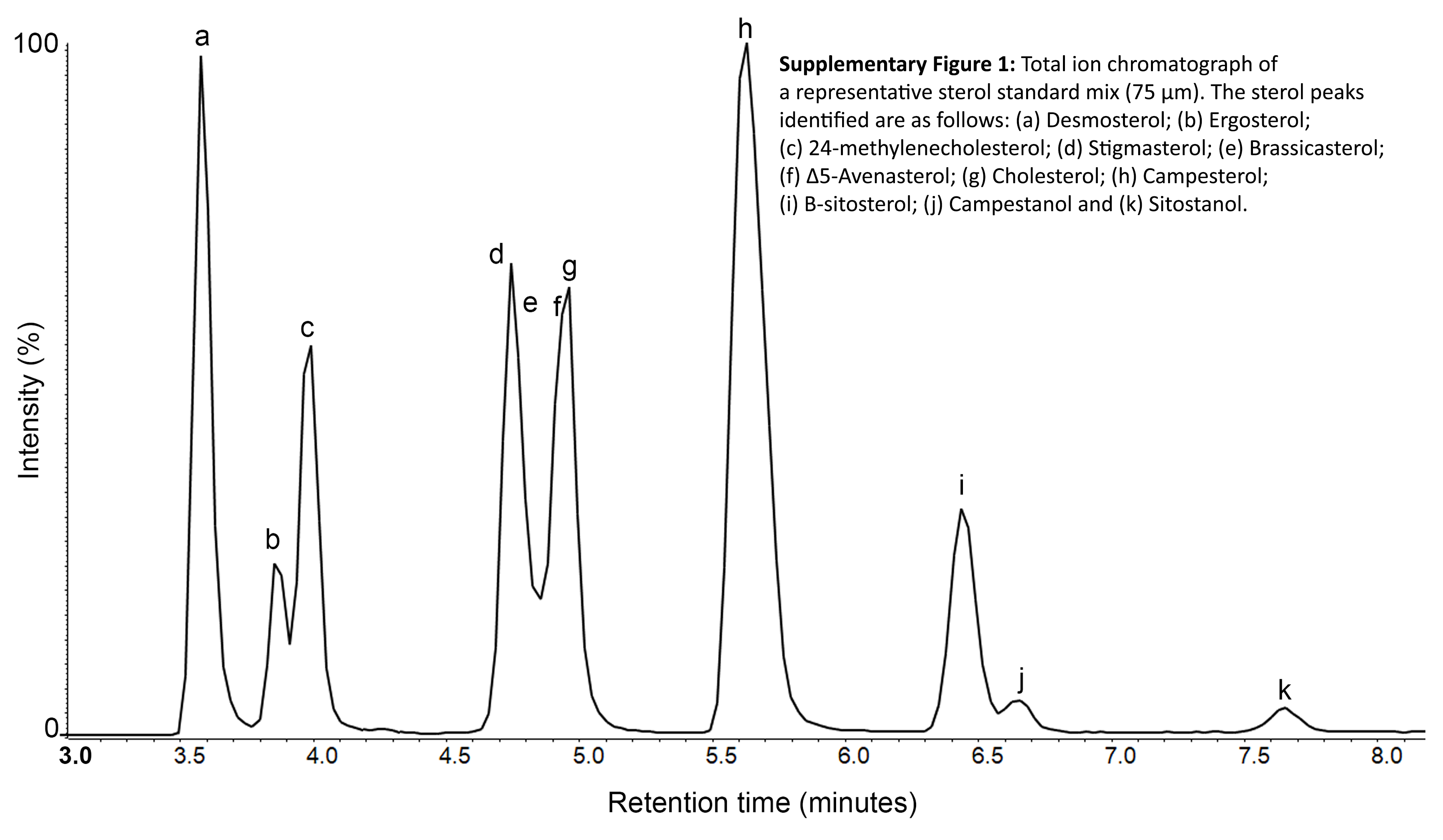

Supplement: Supplementary file 1 — Supplementary material 1 (TIFF 721 kb) [file 11306_2019_1590_MOESM1_ESM.tif]

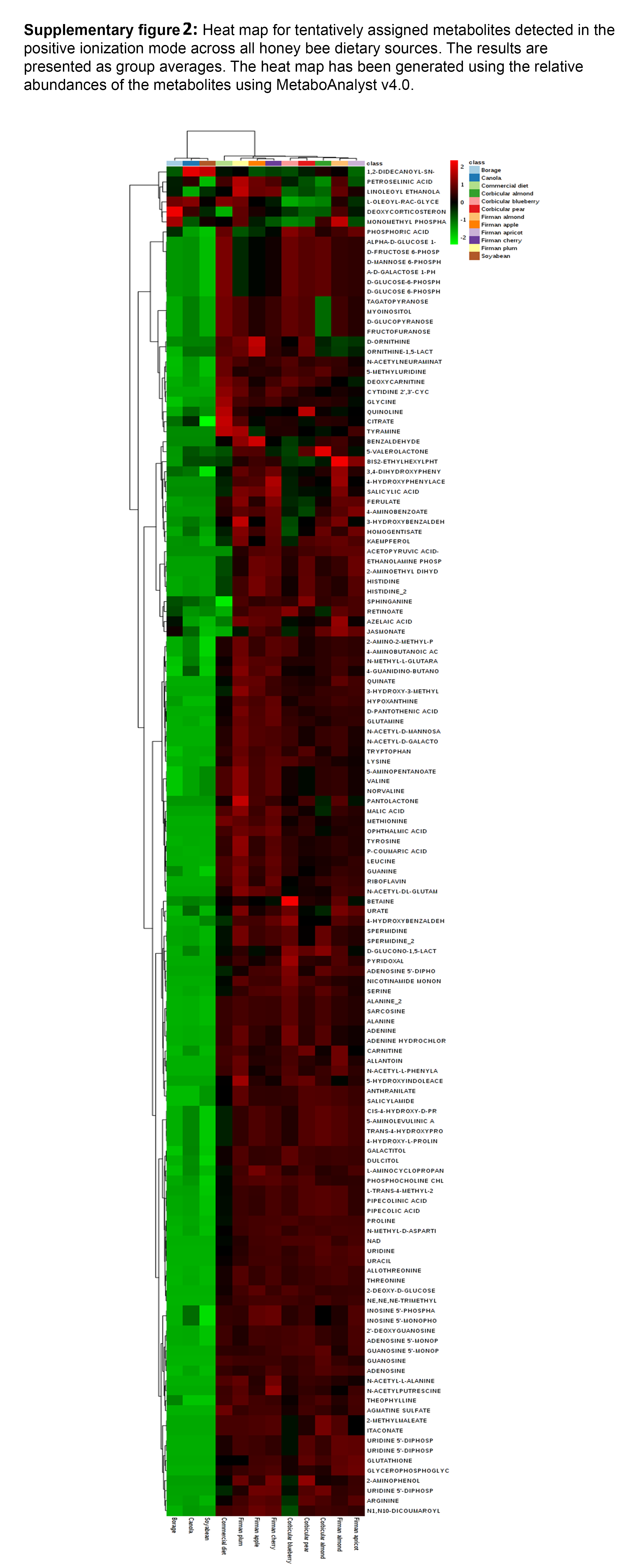

Supplement: Supplementary file 2 — Supplementary material 2 (TIFF 2659 kb) [file 11306_2019_1590_MOESM2_ESM.tif]

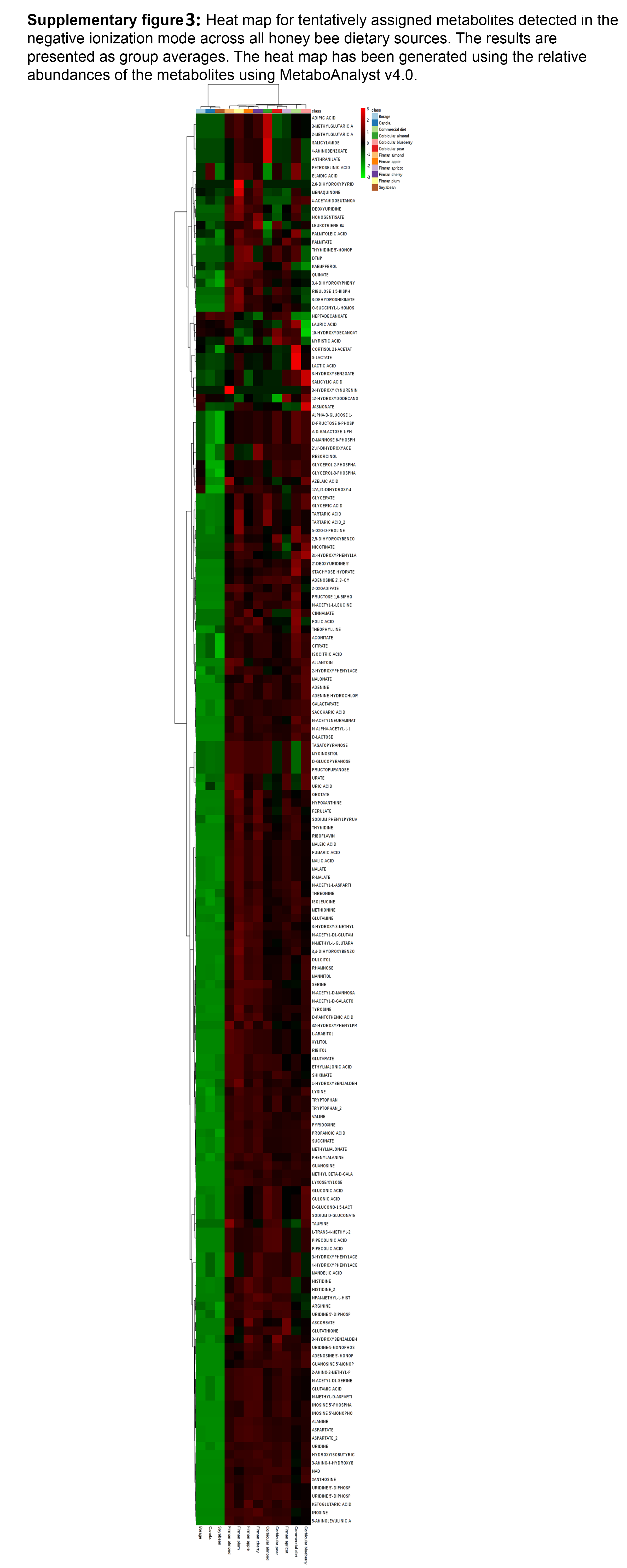

Supplement: Supplementary file 3 — Supplementary material 3 (TIFF 1845 kb) [file 11306_2019_1590_MOESM3_ESM.tif]

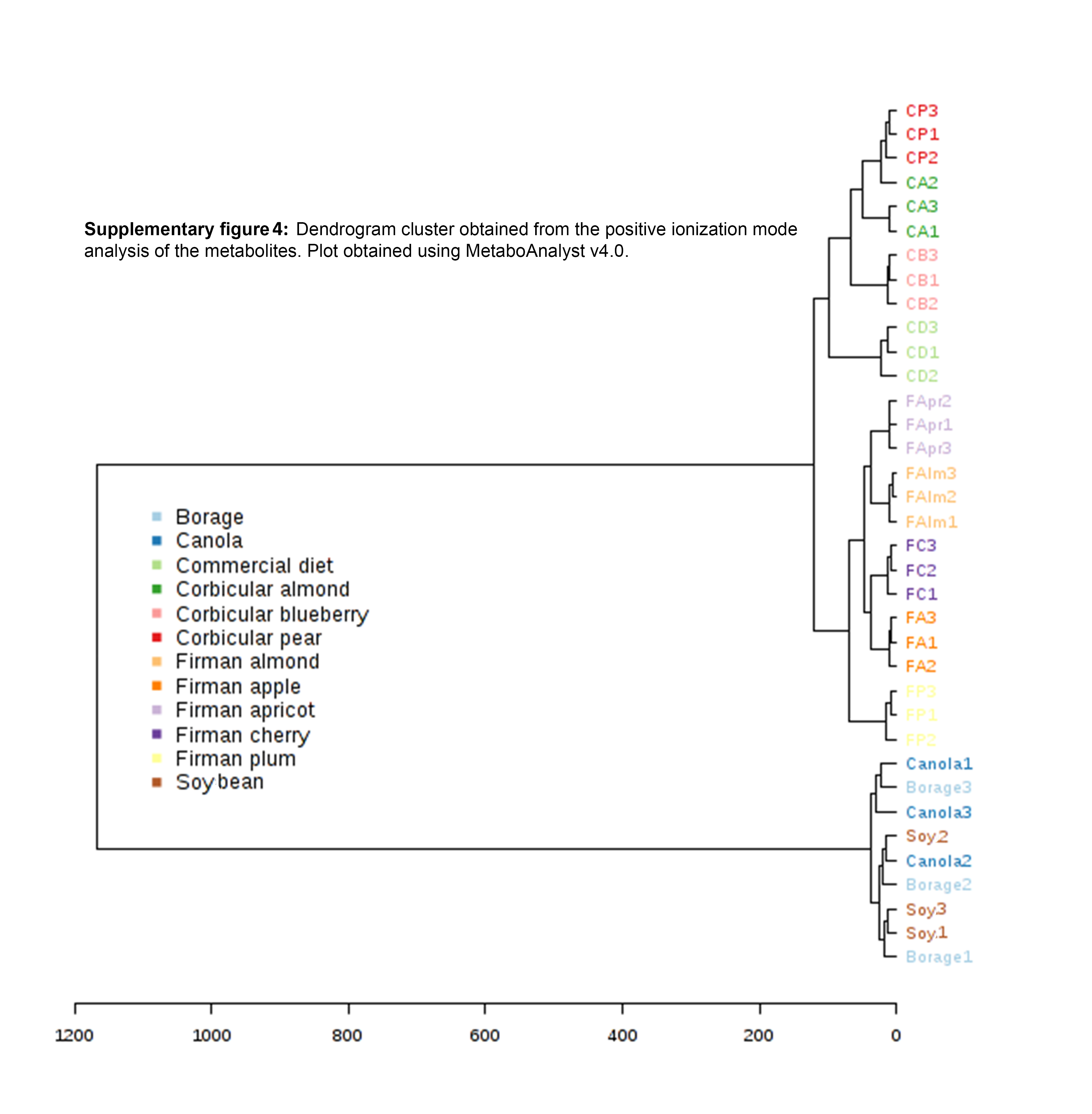

Supplement: Supplementary file 4 — Supplementary material 4 (TIFF 1321 kb) [file 11306_2019_1590_MOESM4_ESM.tif]

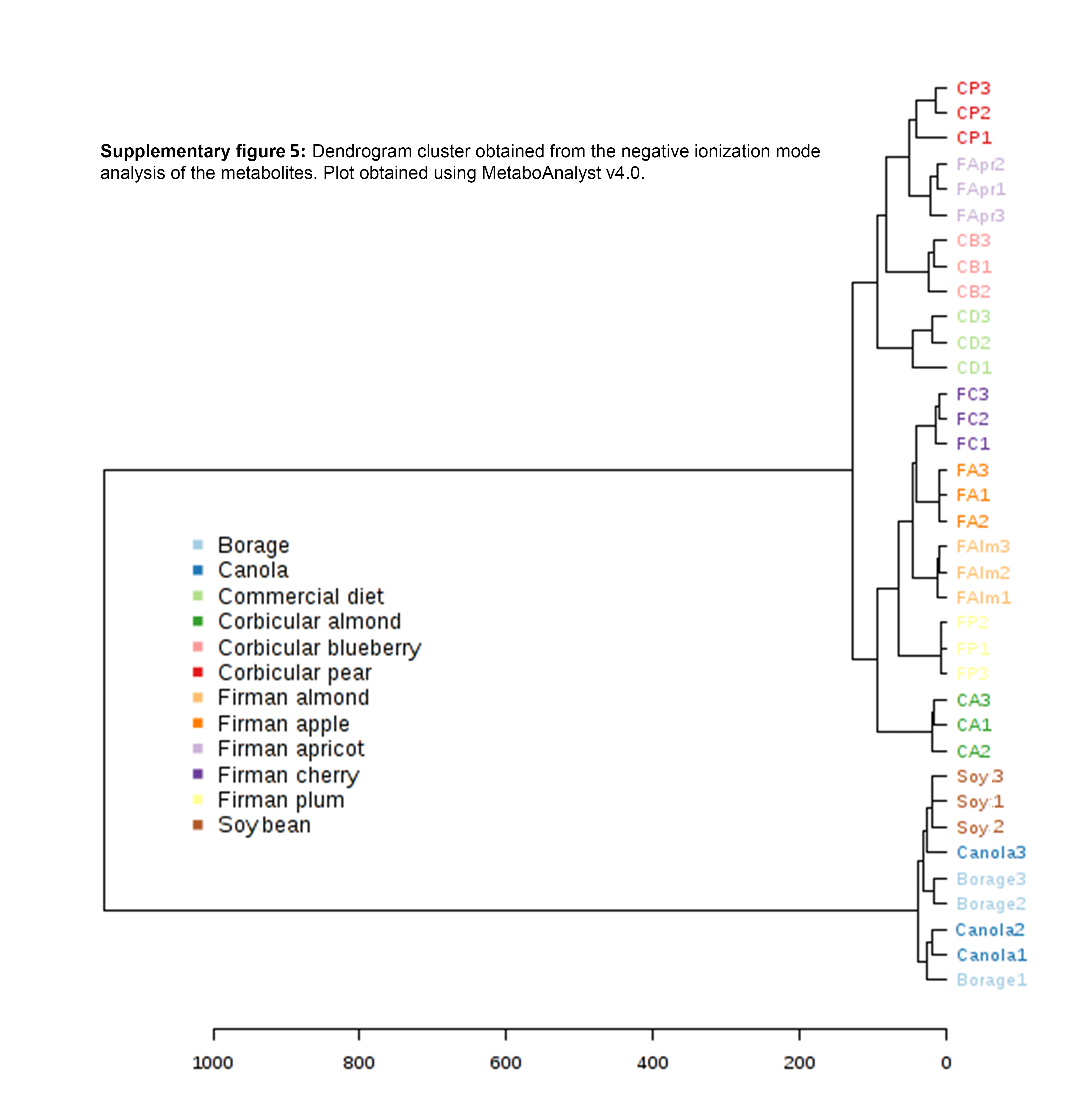

Supplement: Supplementary file 5 — Supplementary material 5 (TIFF 1456 kb) [file 11306_2019_1590_MOESM5_ESM.tif]

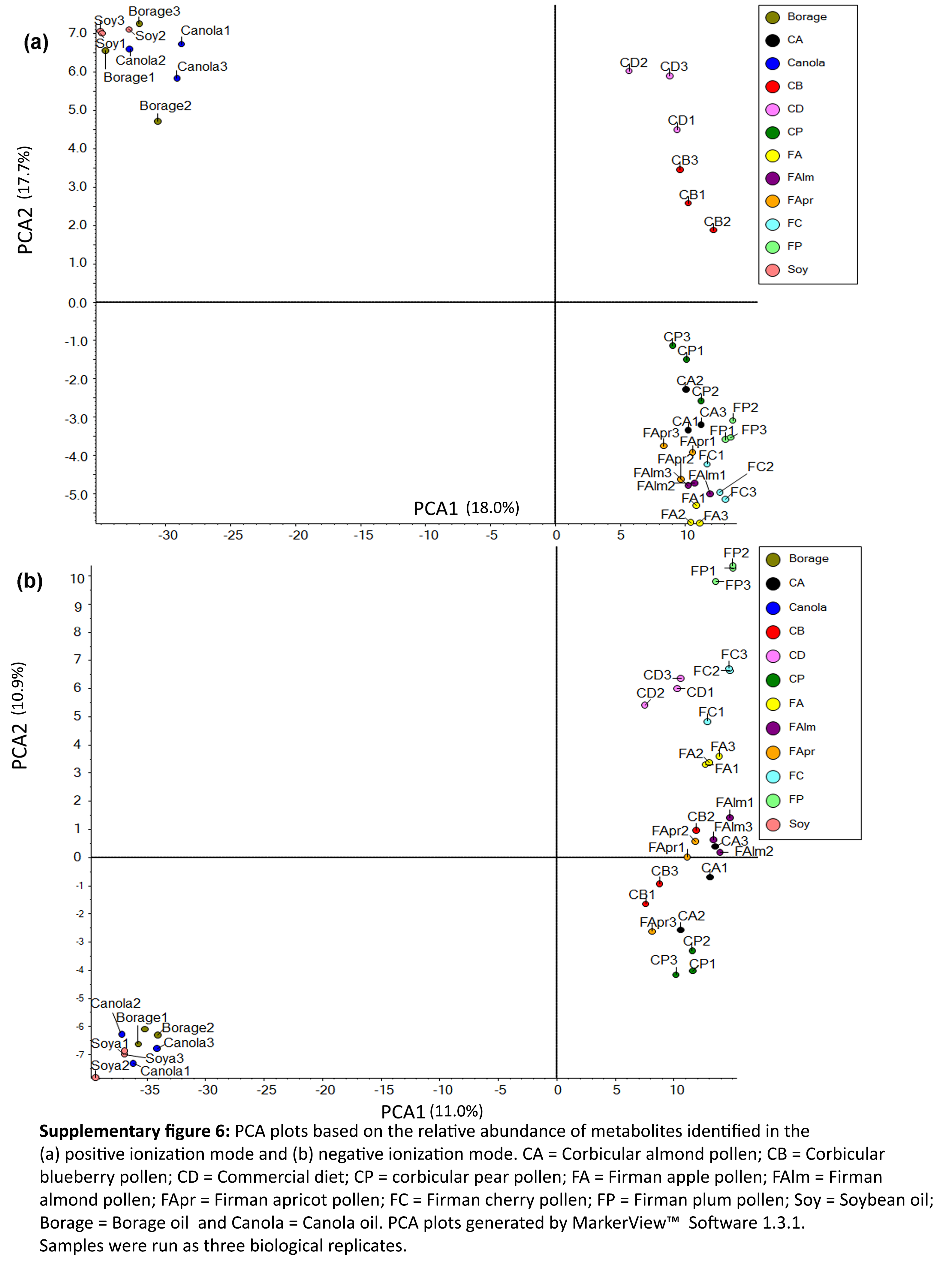

Supplement: Supplementary file 6 — Supplementary material 6 (TIFF 1774 kb) [file 11306_2019_1590_MOESM6_ESM.tif]

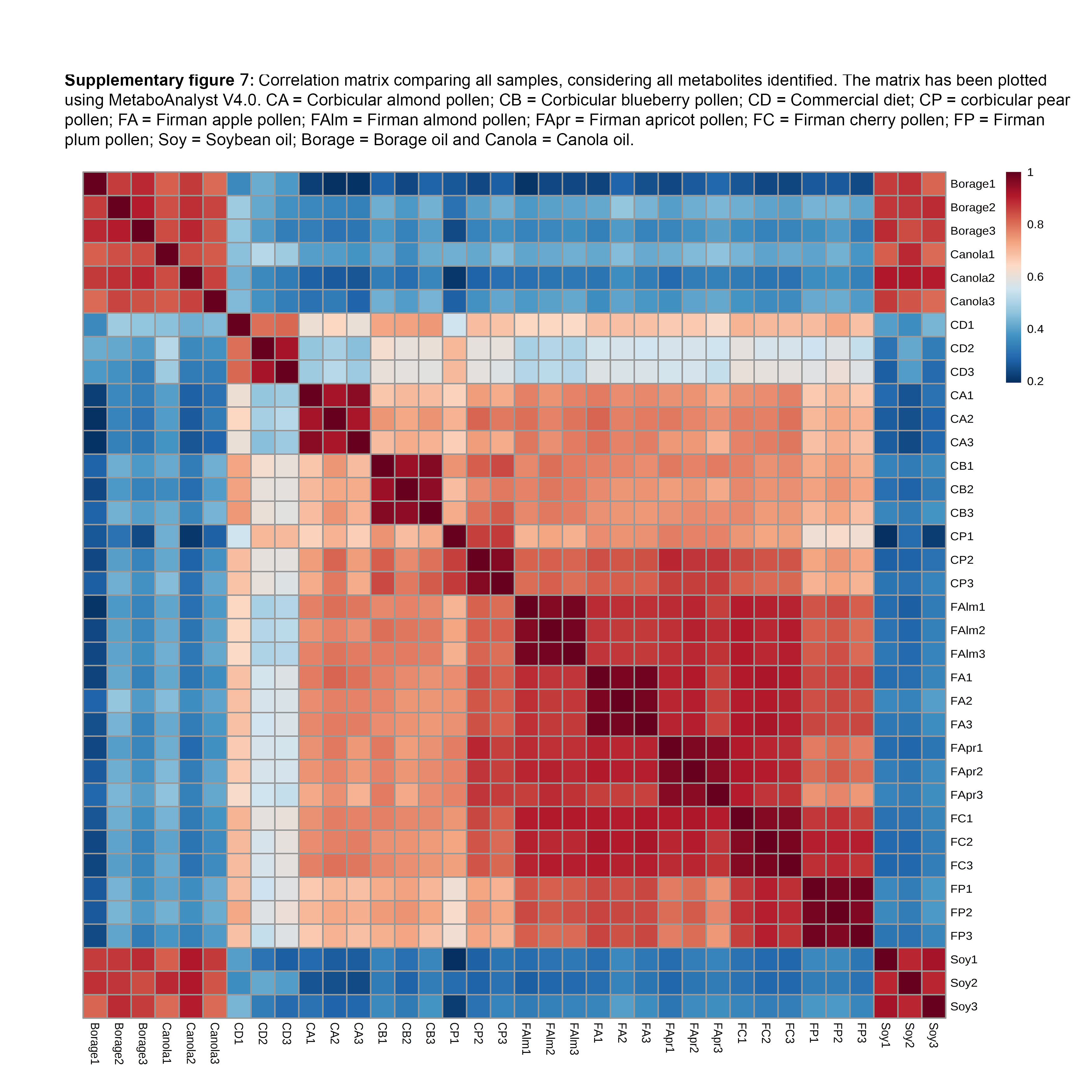

Supplement: Supplementary file 7 — Supplementary material 7 (TIFF 2450 kb) [file 11306_2019_1590_MOESM7_ESM.tif]

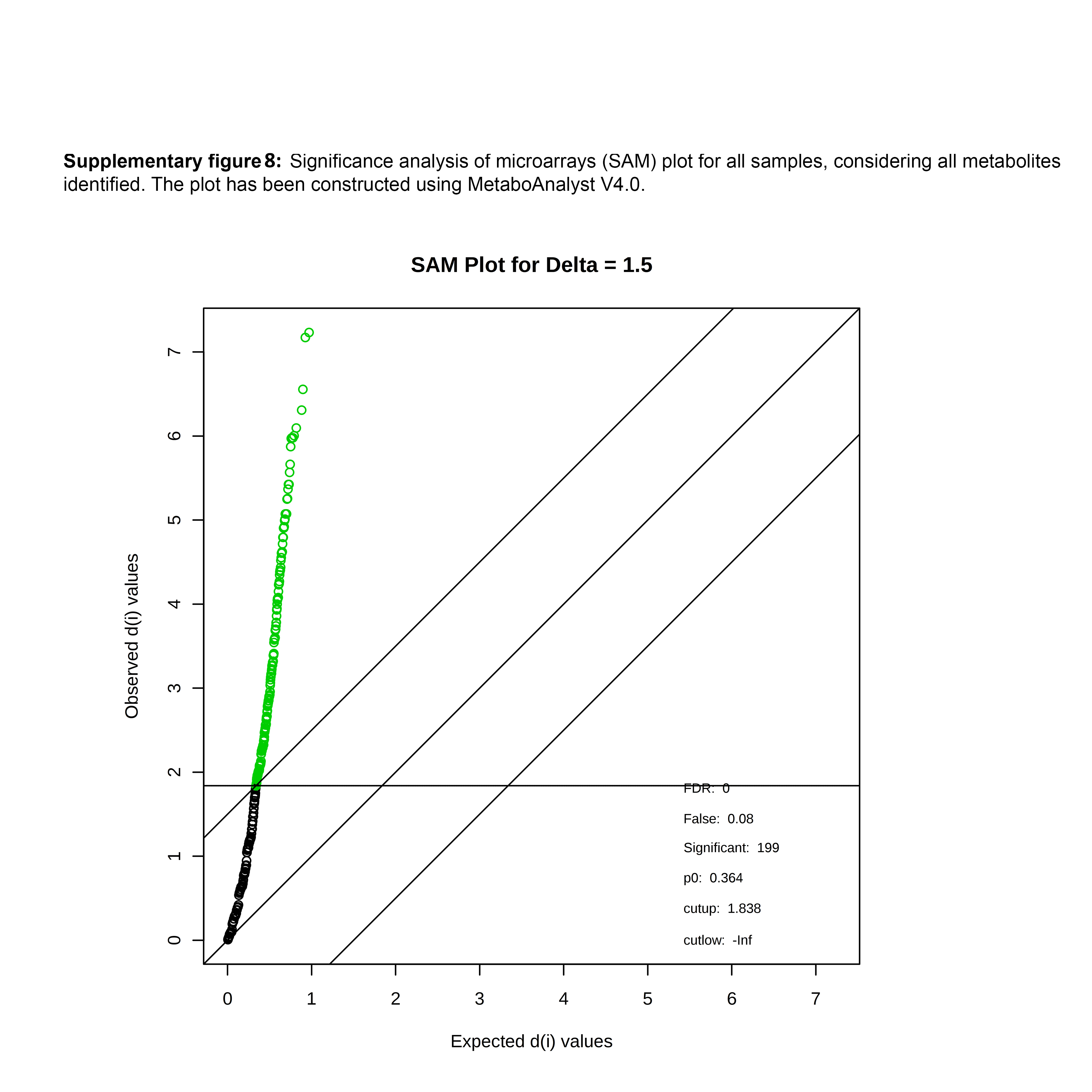

Supplement: Supplementary file 8 — Supplementary material 8 (TIFF 524 kb) [file 11306_2019_1590_MOESM8_ESM.tif]

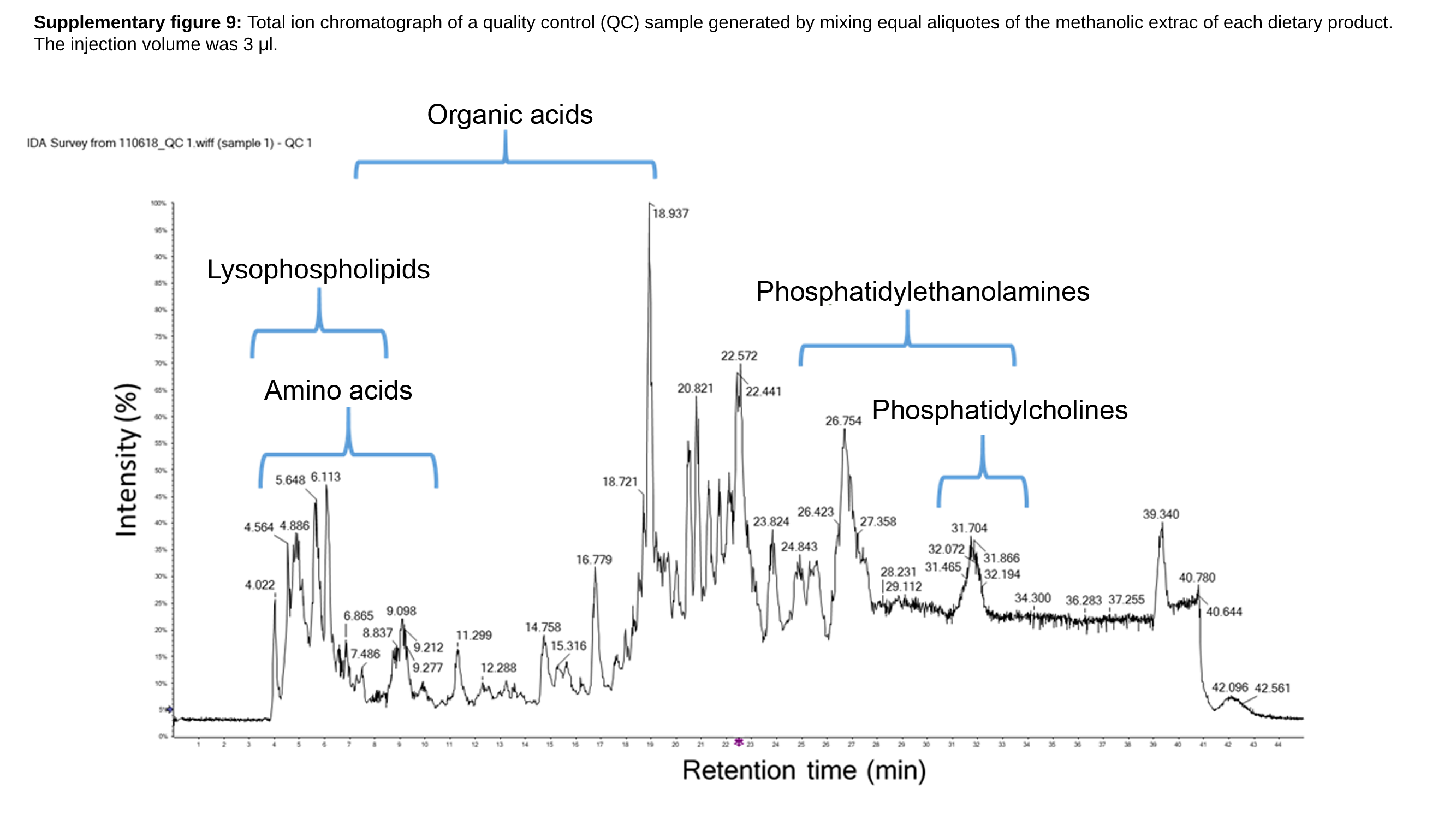

Supplement: Supplementary file 9 — Supplementary material 9 (TIFF 15627 kb) [file 11306_2019_1590_MOESM9_ESM.tiff]
