# Supplementary material for: The omics approach to bee nutritional landscape
Source: Metabolomics. 2019 Sep 20;15(10):127. doi: 10.1007/s11306-019-1590-6 (PMC6753177; doi:10.1007/s11306-019-1590-6)
Supplement: Supplementary file 10 — Supplementary material 10 (DOCX 40 kb) [file 11306_2019_1590_MOESM10_ESM.docx]

**Supplementary table 1:** The table presents the total list of all metabolites putatively identified across all dietary samples. D indicates detected and ND indicates not detected for a particular metabolite for a particular sample.

| *Metabolites* | *Vegetable oils* | | | *Corbicular pollens* | | | *Commercial pollens* | | | | | *Commercial diet* |
| --- | --- | --- | --- | --- | --- | --- | --- | --- | --- | --- | --- | --- |
|  | *Borage* | *Canola* | *Soybean* | *Almond* | *Blueberry* | *Pear* | *Apple* | *Almond* | *Apricot* | *Cherry* | *Plum* |  |
| (R)-MALATE | D | D | D | D | D | D | D | D | D | D | D | D |
| (S)-LACTATE | D | D | D | D | D | D | D | D | D | D | D | D |
| 1,2-DIDECANOYL-SN-GLYCERO-3-PHOSPHOCHOLINE | D | D | D | D | D | D | D | D | D | D | D | D |
| 10-HYDROXYDECANOATE | D | D | D | D | D | D | D | D | D | D | D | D |
| 12-HYDROXYDODECANOIC ACID | D | D | D | D | D | D | D | D | D | D | D | D |
| 17A,21-DIHYDROXY-4-PREGNENE-3,20-DIONE | D | ND | ND | D | D | D | D | D | D | D | D | D |
| 2',4'-DIHYDROXYACETOPHENONE | D | D | D | D | D | D | D | D | D | D | D | D |
| 2,5-DIHYDROXYBENZOATE | ND | ND | ND | D | D | D | D | D | D | D | D | D |
| 2,6-DIHYDROXYPYRIDINE | ND | ND | ND | ND | ND | ND | ND | ND | ND | D | D | ND |
| 2-AMINO-2-METHYL-PROPANOATE | D | D | D | D | D | D | D | D | D | D | D | D |
| 2-AMINO-2-METHYL-PROPANOATE | ND | ND | ND | D | D | D | D | D | D | D | D | D |
| 2-AMINOETHYL DIHYDROGEN PHOSPHATE | ND | ND | ND | D | D | D | D | D | D | D | D | D |
| 2-AMINOPHENOL | ND | ND | ND | D | D | D | D | D | D | D | D | D |
| 2-DEOXY-D-GLUCOSE | ND | ND | ND | D | D | D | D | D | D | D | D | D |
| 2'-DEOXYGUANOSINE 5'-MONOPHOSPHATE | D | D | ND | D | D | D | D | D | D | D | D | D |
| 2'-DEOXYURIDINE 5'-MONOPHOSPHATE | ND | ND | ND | D | D | D | D | D | D | D | D | D |
| 2-HYDROXYPHENYLACETIC ACID | ND | D | ND | D | D | D | D | D | D | D | D | D |
| 2-METHYLGLUTARIC ACID | ND | ND | ND | D | D | ND | D | D | D | D | D | D |
| 2-METHYLMALEATE | ND | ND | ND | D | D | D | D | D | D | D | D | D |
| 2-OXOADIPATE | ND | ND | ND | D | D | D | D | D | D | D | D | D |
| 3(2-HYDROXYPHENYL)PROPANOATE | ND | ND | ND | D | D | D | D | D | D | D | D | D |
| 3(4-HYDROXYPHENYL)LACTATE | ND | ND | ND | D | D | D | D | D | D | D | D | D |
| 3,4-DIHYDROXYBENZOATE | ND | ND | ND | D | D | D | D | D | D | D | D | D |
| 3,4-DIHYDROXYPHENYLACETATE | D | D | D | D | D | D | D | D | D | D | D | D |
| 3,4-DIHYDROXYPHENYLACETATE | D | D | ND | D | D | D | D | D | D | D | D | D |
| 3-AMINO-4-HYDROXYBENZOIC ACID | ND | ND | ND | D | D | D | D | D | D | D | D | D |
| 3-DEHYDROSHIKIMATE | ND | ND | ND | D | D | D | D | D | D | D | D | D |
| 3-HYDROXY-3-METHYLGLUTARATE | ND | ND | ND | D | D | D | D | D | D | D | D | D |
| 3-HYDROXY-3-METHYLGLUTARATE | D | D | D | D | D | D | D | D | D | D | D | D |
| 3-HYDROXYBENZALDEHYDE | ND | D | ND | D | D | D | D | D | D | D | D | D |
| 3-HYDROXYBENZALDEHYDE | D | D | D | D | D | D | D | D | D | D | D | D |
| 3-HYDROXYBENZOATE | D | D | D | D | D | D | D | D | D | D | D | D |
| 3-HYDROXYKYNURENINE | ND | ND | ND | ND | D | ND | ND | D | ND | ND | ND | ND |
| 3-HYDROXYPHENYLACETATE | ND | D | ND | D | D | D | D | D | D | D | D | D |
| 3-METHYLGLUTARIC ACID | ND | ND | ND | D | D | ND | D | D | D | D | D | D |
| 4-ACETAMIDOBUTANOATE | ND | D | ND | D | D | ND | D | D | ND | D | D | D |
| 4-AMINOBENZOATE | ND | ND | ND | D | D | D | D | D | D | D | D | D |
| 4-AMINOBENZOATE | ND | ND | ND | D | ND | D | D | D | D | D | D | D |
| 4-AMINOBUTANOIC ACID | D | D | D | D | D | D | D | D | D | D | D | D |
| 4-GUANIDINO-BUTANOATE | ND | D | ND | D | D | D | D | D | D | D | D | D |
| 4-HYDROXYBENZALDEHYDE | ND | ND | D | D | D | D | D | D | D | D | D | D |
| 4-HYDROXYBENZALDEHYDE | D | D | D | D | D | D | D | D | D | D | D | D |
| 4-HYDROXY-L-PROLINE | D | D | ND | D | D | D | D | D | D | D | D | D |
| 4-HYDROXYPHENYLACETATE | ND | ND | ND | D | D | D | D | D | D | D | D | D |
| 4-HYDROXYPHENYLACETATE | ND | D | ND | D | D | D | D | D | D | D | D | D |
| 5-AMINOLEVULINIC ACID | D | D | ND | D | D | D | D | D | D | D | D | D |
| 5-AMINOLEVULINIC ACID | ND | ND | ND | D | D | D | D | D | D | D | D | D |
| 5-AMINOPENTANOATE | D | D | D | D | D | D | D | D | D | D | D | D |
| 5-HYDROXYINDOLEACETATE | ND | ND | ND | D | D | D | D | D | D | D | D | D |
| 5-METHYLURIDINE | D | D | D | D | D | D | D | D | D | D | D | D |
| 5-OXO-D-PROLINE | D | D | D | D | D | D | D | D | D | D | D | D |
| 5-VALEROLACTONE | D | D | D | D | D | D | D | D | D | D | D | D |
| ACETOPYRUVIC ACID-LIKE | ND | ND | ND | D | D | D | D | D | D | D | D | D |
| ACONITATE | D | D | D | D | D | D | D | D | D | D | D | D |
| ADENINE | D | D | D | D | D | D | D | D | D | D | D | D |
| ADENINE HYDROCHLORIDE | D | D | D | D | D | D | D | D | D | D | D | D |
| ADENOSINE | ND | D | ND | D | D | D | D | D | D | D | D | D |
| ADENOSINE 2',3'-CYCLIC MONOPHOSPHATE | ND | ND | ND | D | D | D | D | D | D | D | D | D |
| ADENOSINE 5'-DIPHOSPHORIBOSE | ND | ND | ND | D | D | D | D | D | D | D | D | D |
| ADENOSINE 5'-MONOPHOSPHATE | D | D | ND | D | D | D | D | D | D | D | D | D |
| A-D-GALACTOSE 1-PHOSPHATE DIPOTASSIUM SALT PENTAHYDRATE | D | D | ND | D | D | D | D | D | D | D | D | D |
| ADIPIC ACID | ND | ND | ND | D | D | ND | D | D | D | D | D | D |
| AGMATINE SULFATE | ND | ND | ND | D | D | D | D | D | D | D | D | D |
| ALANINE | D | D | D | D | D | D | D | D | D | D | D | D |
| ALLANTOIN | ND | ND | ND | D | D | D | D | D | D | D | D | D |
| ALLOTHREONINE | D | D | D | D | D | D | D | D | D | D | D | D |
| ALPHA-D-GLUCOSE 1-PHOSPHATE | D | D | ND | D | D | D | D | D | D | D | D | D |
| ANTHRANILATE | ND | ND | D | D | D | D | D | D | D | D | D | D |
| ARGININE | D | D | ND | D | D | D | D | D | D | D | D | D |
| ASCORBATE | ND | ND | ND | D | D | D | D | D | D | D | D | D |
| ASPARTATE | D | D | D | D | D | D | D | D | D | D | D | D |
| AZELAIC ACID | D | D | D | D | D | D | D | D | D | D | D | D |
| BENZALDEHYDE | ND | ND | ND | D | D | D | D | D | D | D | D | D |
| BETAINE | D | D | D | D | D | D | D | D | D | D | D | D |
| BIS(2-ETHYLHEXYL)PHTHALATE | D | D | D | D | D | D | D | D | D | D | D | D |
| CARNITINE | D | ND | D | D | D | D | D | D | D | D | D | D |
| CINNAMATE | ND | ND | ND | D | D | D | D | D | D | D | D | D |
| CIS-4-HYDROXY-D-PROLINE | D | D | ND | D | D | D | D | D | D | D | D | D |
| CITRATE | D | D | D | D | D | D | D | D | D | D | D | D |
| CORTISOL 21-ACETATE | D | D | D | D | D | D | D | D | D | D | D | D |
| CYTIDINE 2',3'-CYCLIC MONOPHOSPHATE | ND | ND | ND | D | D | D | D | D | D | D | D | D |
| DEOXYCARNITINE | ND | D | ND | D | D | D | D | D | D | D | D | D |
| DEOXYCORTICOSTERONE | D | D | D | D | D | D | D | D | D | D | D | D |
| DEOXYURIDINE | ND | ND | ND | D | D | ND | D | D | D | D | D | D |
| D-FRUCTOSE 6-PHOSPHATE | ND | ND | D | D | D | D | D | D | D | D | D | D |
| D-GLUCONO-1,5-LACTONE | ND | D | ND | D | D | D | D | D | D | D | D | D |
| D-GLUCOPYRANOSE | ND | ND | ND | D | D | D | D | D | D | D | D | D |
| D-GLUCOSE 6-PHOSPHATE | D | D | ND | D | D | D | D | D | D | D | D | D |
| D-LACTOSE | ND | ND | ND | D | D | D | D | D | D | D | D | D |
| D-MANNOSE 6-PHOSPHATE | D | D | ND | D | D | D | D | D | D | D | D | D |
| D-ORNITHINE | D | D | D | D | D | D | D | D | D | D | D | D |
| D-PANTOTHENIC ACID | ND | ND | ND | D | D | D | D | D | D | D | D | D |
| DTMP | ND | ND | ND | D | ND | D | D | D | D | D | D | D |
| DULCITOL | D | D | ND | D | D | D | D | D | D | D | D | D |
| ELAIDIC ACID | D | D | D | D | D | D | D | D | D | D | D | D |
| ETHANOLAMINE PHOSPHATE | ND | ND | ND | D | D | D | D | D | D | D | D | D |
| ETHYLMALONIC ACID | ND | ND | D | D | D | D | D | D | D | D | D | D |
| FERULATE | D | D | D | D | D | D | D | D | D | D | D | D |
| FOLIC ACID | ND | ND | ND | D | D | D | D | D | D | D | D | D |
| FRUCTOFURANOSE | ND | D | ND | D | D | D | D | D | D | D | D | D |
| FRUCTOSE 1,6-BIPHOSPHATE | ND | ND | ND | D | D | D | D | D | D | D | D | D |
| FUMARIC ACID | ND | ND | ND | D | D | D | D | D | D | D | D | D |
| GALACTARATE | ND | D | ND | D | D | D | D | D | D | D | D | D |
| GALACTITOL | ND | D | ND | D | D | D | D | D | D | D | D | D |
| GLUCONIC ACID | D | D | D | D | D | D | D | D | D | D | D | D |
| GLUTAMIC ACID | D | D | D | D | D | D | D | D | D | D | D | D |
| GLUTAMINE | D | D | D | D | D | D | D | D | D | D | D | D |
| GLUTARATE | ND | ND | D | D | D | D | D | D | D | D | D | D |
| GLUTATHIONE | ND | ND | ND | D | D | D | D | D | D | D | D | D |
| GLYCERATE | D | D | D | D | D | D | D | D | D | D | D | D |
| GLYCERIC ACID | D | D | D | D | D | D | D | D | D | D | D | D |
| GLYCEROL 2-PHOSPHATE | D | ND | ND | D | D | D | D | D | D | D | D | D |
| GLYCEROL-3-PHOSPHATE | D | D | ND | D | D | D | D | D | D | D | D | D |
| GLYCEROPHOSPHOGLYCEROL | ND | ND | ND | D | D | D | D | D | D | D | D | D |
| GLYCINE | D | D | D | D | D | D | D | D | D | D | D | D |
| GUANINE | D | D | ND | D | D | D | D | D | D | D | D | D |
| GUANOSINE | ND | ND | ND | D | D | D | D | D | D | D | D | D |
| GUANOSINE 5'-MONOPHOSPHATE | ND | ND | ND | D | D | D | D | D | D | D | D | D |
| GULONIC ACID | ND | D | ND | D | D | D | D | D | D | D | D | D |
| HEPTADECANOATE | D | D | D | D | D | D | D | D | D | D | D | D |
| HISTIDINE | D | D | D | D | D | D | D | D | D | D | D | D |
| HOMOGENTISATE | ND | D | ND | D | D | D | D | D | D | D | D | D |
| HYDROXYISOBUTYRIC ACID | ND | ND | ND | D | D | D | D | D | D | D | D | D |
| HYPOXANTHINE | D | ND | ND | D | D | D | D | D | D | D | D | D |
| INOSINE | ND | ND | ND | D | D | D | D | D | D | D | D | D |
| INOSINE 5'-MONOPHOSPHATE | D | D | D | D | D | D | D | D | D | D | D | D |
| INOSINE 5'-PHOSPHATE | D | D | D | D | D | D | D | D | D | D | D | D |
| ISOCITRIC ACID | D | D | D | D | D | D | D | D | D | D | D | D |
| ISOLEUCINE | D | ND | ND | D | D | D | D | D | D | D | D | D |
| ITACONATE | ND | ND | ND | D | D | D | D | D | D | D | D | D |
| JASMONATE | D | D | D | D | D | D | D | D | D | D | D | D |
| KAEMPFEROL | D | D | ND | D | D | D | D | D | D | D | D | D |
| KETOGLUTARIC ACID | ND | ND | ND | D | D | D | D | D | D | D | D | D |
| LACTIC ACID | D | D | D | D | D | D | D | D | D | D | D | D |
| L-AMINOCYCLOPROPANE-1-CARBOXYLATE | D | D | D | D | D | D | D | D | D | D | D | D |
| L-ARABITOL | ND | ND | ND | D | D | D | D | D | D | D | D | D |
| LAURIC ACID | D | D | D | D | D | D | D | D | D | D | D | D |
| LEUCINE | D | D | D | D | D | D | D | D | D | D | D | D |
| LEUKOTRIENE B4 | D | D | D | D | D | D | D | D | D | D | D | D |
| LINOLEOYL ETHANOLAMIDE | D | D | D | D | D | D | D | D | D | D | D | D |
| L-OLEOYL-RAC-GLYCEROL | D | D | D | D | D | D | D | D | D | D | D | D |
| L-TRANS-4-METHYL-2-PYRROLIDINECARBOXYLIC ACID | D | D | D | D | D | D | D | D | D | D | D | D |
| LYSINE | D | D | D | D | D | D | D | D | D | D | D | D |
| LYXOSE/XYLOSE | ND | ND | ND | D | D | D | D | D | D | D | D | D |
| MALATE | D | D | D | D | D | D | D | D | D | D | D | D |
| MALEIC ACID | ND | ND | ND | D | D | D | D | D | D | D | D | D |
| MALIC ACID | ND | ND | ND | D | D | D | D | D | D | D | D | D |
| MALONATE | ND | ND | D | D | D | D | D | D | D | D | D | D |
| MANDELIC ACID | ND | D | ND | D | D | D | D | D | D | D | D | D |
| MANNITOL | D | D | D | D | D | D | D | D | D | D | D | D |
| MENAQUINONE | D | D | D | D | D | D | D | D | D | D | D | D |
| METHIONINE | ND | ND | ND | D | D | D | D | D | D | D | D | D |
| METHYL BETA-D-GALACTOSIDE | ND | ND | ND | D | D | D | D | D | D | D | D | D |
| METHYLMALONATE | D | D | D | D | D | D | D | D | D | D | D | D |
| MONOMETHYL PHOSPHATE | D | D | D | D | D | D | D | D | D | D | D | D |
| MYOINOSITOL | ND | ND | ND | D | D | D | D | D | D | D | D | D |
| MYRISTIC ACID | D | D | D | D | D | D | D | D | D | D | D | D |
| N ALPHA-ACETYL-L-LYSINE | ND | ND | ND | D | D | D | D | D | D | D | D | D |
| N(PAI)-METHYL-L-HISTIDINE | ND | ND | ND | D | D | D | D | D | D | D | D | D |
| N1,N10-DICOUMAROYLSPERMIDINE | D | D | D | D | D | D | D | D | D | D | D | D |
| N-ACETYL-D-GALACTOSAMINE | ND | ND | ND | D | D | D | D | D | D | D | D | D |
| N-ACETYL-DL-GLUTAMIC ACID | ND | ND | ND | D | D | D | D | D | D | D | D | D |
| N-ACETYL-DL-SERINE | D | D | D | D | D | D | D | D | D | D | D | D |
| N-ACETYL-D-MANNOSAMINE | ND | ND | ND | D | D | D | D | D | D | D | D | D |
| N-ACETYL-L-ALANINE | ND | ND | ND | D | D | D | D | D | D | D | D | D |
| N-ACETYL-L-ASPARTIC ACID | ND | ND | ND | D | D | D | D | D | D | D | D | D |
| N-ACETYL-L-LEUCINE | ND | ND | ND | D | D | D | D | D | D | D | D | D |
| N-ACETYL-L-PHENYLALANINE | ND | ND | ND | D | D | D | D | D | D | D | D | D |
| N-ACETYLNEURAMINATE | ND | D | ND | D | D | D | D | D | D | D | D | D |
| N-ACETYLPUTRESCINE | ND | ND | ND | D | D | D | D | D | D | D | D | D |
| NAD | ND | ND | ND | D | D | D | D | D | D | D | D | D |
| NE,NE,NE-TRIMETHYLLYSINE | ND | ND | ND | D | D | D | D | D | D | D | D | D |
| NICOTINAMIDE MONONUCLEOTIDE | ND | ND | ND | D | D | D | D | D | D | D | D | D |
| NICOTINATE | ND | ND | ND | D | D | D | D | D | D | D | D | D |
| N-METHYL-D-ASPARTIC ACID | D | D | D | D | D | D | D | D | D | D | D | D |
| N-METHYL-L-GLUTARATE | ND | D | ND | D | D | D | D | D | D | D | D | D |
| NORVALINE | D | D | D | D | D | D | D | D | D | D | D | D |
| OPHTHALMIC ACID | ND | ND | ND | D | D | D | D | D | D | D | D | D |
| ORNITHINE-1,5-LACTUM | D | D | D | D | D | D | D | D | D | D | D | D |
| OROTATE | ND | ND | ND | D | D | D | D | D | D | D | D | D |
| O-SUCCINYL-L-HOMOSERINE | ND | ND | ND | D | D | D | D | D | D | D | D | D |
| PALMITATE | D | D | D | D | D | D | D | D | D | D | D | D |
| PALMITOLEIC ACID | D | D | D | D | D | D | D | D | D | D | D | D |
| PANTOLACTONE | ND | ND | ND | D | D | D | D | D | D | D | D | D |
| P-COUMARIC ACID | D | D | D | D | D | D | D | D | D | D | D | D |
| PETROSELINIC ACID | D | D | D | D | D | D | D | D | D | D | D | D |
| PHENYLALANINE | D | ND | ND | D | D | D | D | D | D | D | D | D |
| PHOSPHOCHOLINE CHLORIDE | D | D | D | D | D | D | D | D | D | D | D | D |
| PHOSPHORIC ACID | D | D | D | D | D | D | D | D | D | D | D | D |
| PIPECOLIC ACID | ND | ND | ND | D | D | D | D | D | D | D | D | D |
| PIPECOLINIC ACID | ND | ND | ND | D | D | D | D | D | D | D | D | D |
| PROLINE | D | D | D | D | D | D | D | D | D | D | D | D |
| PROPANOIC ACID | D | D | D | D | D | D | D | D | D | D | D | D |
| PYRIDOXAL | ND | ND | ND | D | D | D | D | D | D | D | D | D |
| PYRIDOXINE | ND | ND | ND | D | D | D | D | D | D | D | D | D |
| QUINATE | ND | ND | ND | D | D | D | D | D | D | D | D | D |
| QUINOLINE | D | D | D | D | D | D | D | D | D | D | D | D |
| RESORCINOL | D | D | D | D | D | D | D | D | D | D | D | D |
| RETINOATE | D | D | D | D | D | D | D | D | D | D | D | D |
| RHAMNOSE | D | D | D | D | D | D | D | D | D | D | D | D |
| RIBITOL | ND | ND | ND | D | D | D | D | D | D | D | D | D |
| RIBOFLAVIN | ND | ND | ND | D | D | D | D | D | D | D | D | D |
| RIBULOSE 1,5-BISPHOSPHATE | ND | ND | ND | D | D | D | D | D | D | D | D | D |
| SACCHARIC ACID | ND | D | ND | D | D | D | D | D | D | D | D | D |
| SALICYLAMIDE | ND | ND | D | D | D | D | D | D | D | D | D | D |
| SALICYLIC ACID | ND | ND | ND | D | D | D | D | D | D | D | D | D |
| SARCOSINE | D | D | D | D | D | D | D | D | D | D | D | D |
| SERINE | D | D | D | D | D | D | D | D | D | D | D | D |
| SHIKIMATE | ND | ND | ND | D | D | D | D | D | D | D | D | D |
| SODIUM D-GLUCONATE | ND | D | ND | D | D | D | D | D | D | D | D | D |
| SODIUM PHENYLPYRUVATE | D | ND | ND | D | D | D | D | D | D | D | D | D |
| SPERMIDINE | D | D | D | D | D | D | D | D | D | D | D | D |
| SPHINGANINE | D | D | D | D | D | D | D | D | D | D | D | D |
| STACHYOSE HYDRATE | ND | ND | ND | D | D | D | D | D | D | D | D | D |
| SUCCINATE | D | D | D | D | D | D | D | D | D | D | D | D |
| TAGATOPYRANOSE | ND | D | ND | D | D | D | D | D | D | D | D | D |
| TARTARIC ACID | D | D | D | D | D | D | D | D | D | D | D | D |
| TAURINE | ND | ND | ND | D | D | D | D | D | D | D | D | D |
| THEOPHYLLINE | D | ND | ND | D | D | D | D | D | D | D | D | D |
| THREONINE | D | D | D | D | D | D | D | D | D | D | D | D |
| THYMIDINE | ND | ND | ND | D | D | D | D | D | D | D | D | D |
| THYMIDINE 5'-MONOPHOSPHATE | ND | ND | ND | D | ND | D | D | D | D | D | D | D |
| TRANS-4-HYDROXYPROLINE | D | D | ND | D | D | D | D | D | D | D | D | D |
| TRYPTOPHAN | D | D | D | D | D | D | D | D | D | D | D | D |
| TYRAMINE | ND | ND | ND | D | D | D | D | D | D | D | D | D |
| TYROSINE | D | D | D | D | D | D | D | D | D | D | D | D |
| URACIL | ND | ND | ND | D | D | D | D | D | D | D | D | D |
| URATE | ND | D | ND | D | D | D | D | D | D | D | D | D |
| URIC ACID | D | D | D | D | D | D | D | D | D | D | D | D |
| URIDINE | ND | ND | ND | D | D | D | D | D | D | D | D | D |
| URIDINE 5'-DIPHOSPHATE | ND | ND | ND | D | D | D | D | D | D | D | D | D |
| URIDINE 5'-DIPHOSPHOGALACTOSE | D | D | ND | D | D | D | D | D | D | D | D | D |
| URIDINE 5'-DIPHOSPHO-N-ACETYLGALACTOSAMINE | ND | ND | ND | D | D | D | D | D | D | D | D | D |
| URIDINE 5'-DIPHOSPHO-N-ACETYLGLUCOSAMINE | ND | ND | ND | D | D | D | D | D | D | D | D | D |
| URIDINE-5-MONOPHOSPHATE | ND | ND | ND | D | D | D | D | D | D | D | D | D |
| VALINE | D | D | D | D | D | D | D | D | D | D | D | D |
| XANTHOSINE | ND | ND | ND | D | D | D | D | D | D | D | D | D |
| XYLITOL | ND | ND | ND | D | D | D | D | D | D | D | D | D |
