# Supplementary material for: The omics approach to bee nutritional landscape
Source: Metabolomics. 2019 Sep 20;15(10):127. doi: 10.1007/s11306-019-1590-6 (PMC6753177; doi:10.1007/s11306-019-1590-6)
Supplement: Supplementary file 11 — Supplementary material 11 (PDF 117 kb) [file 11306_2019_1590_MOESM11_ESM.pdf]

FA: Firman Apple

FApr: Firman apricot

FAlm: Firman almond

FC: Firman cherry

FP: Firman plum

CA: Corbicular almond

CB: Corbicular blueberry

CP: corbicular pear

CD: Commercial diets (Mann Lake UltraBee)

Soy: Soybean oil

Borage: Borage oil

Canola: Canola oil

24-methylenecholesterol

```
            Df Sum Sq Mean Sq F value Pr(>F)    
Samples     11 20.226  1.8387   134.3 <2e-16 ***
Residuals   24  0.329  0.0137                   
---
Signif. codes:  0 '***' 0.001 '**' 0.01 '*' 0.05 '.' 0.1 ' ' 1


Simultaneous Tests for General Linear Hypotheses

Multiple Comparisons of Means: Tukey Contrasts

Linear Hypotheses:
                      Estimate Std. Error t value Pr(>|t|)    
CA - Borage == 0      0.525678   0.095536   5.502    <0.01 ***
Canola - Borage == 0 -0.902906   0.095536  -9.451    <0.01 ***
CB - Borage == 0      0.424375   0.095536   4.442    <0.01 ** 
CD - Borage == 0     -1.712752   0.095536 -17.928    <0.01 ***
CP - Borage == 0      0.406742   0.095536   4.257   0.0113 *  
FA - Borage == 0      0.446242   0.095536   4.671    <0.01 ** 
FAlm - Borage == 0    0.483571   0.095536   5.062    <0.01 ** 
FApr - Borage == 0    0.572418   0.095536   5.992    <0.01 ***
FC - Borage == 0      0.490215   0.095536   5.131    <0.01 ** 
FP - Borage == 0      0.250671   0.095536   2.624   0.3195    
Soy - Borage == 0    -1.144388   0.095536 -11.979    <0.01 ***
Canola - CA == 0     -1.428583   0.095536 -14.953    <0.01 ***
CB - CA == 0         -0.101302   0.095536  -1.060   0.9938    
CD - CA == 0         -2.238429   0.095536 -23.430    <0.01 ***
CP - CA == 0         -0.118935   0.095536  -1.245   0.9788    
FA - CA == 0         -0.079436   0.095536  -0.831   0.9993    
FAlm - CA == 0       -0.042107   0.095536  -0.441   1.0000    
FApr - CA == 0        0.046741   0.095536   0.489   1.0000    
```

```
FC - CA == 0         -0.035463   0.095536  -0.371   1.0000
FP - CA == 0         -0.275006   0.095536  -2.879   0.2100
Soy - CA == 0        -1.670066   0.095536 -17.481    <0.01 ***
CB - Canola == 0      1.327281   0.095536  13.893    <0.01 ***
CD - Canola == 0     -0.809846   0.095536  -8.477    <0.01 ***
CP - Canola == 0      1.309648   0.095536  13.708    <0.01 ***
FA - Canola == 0      1.349148   0.095536  14.122    <0.01 ***
FAlm - Canola == 0    1.386477   0.095536  14.513    <0.01 ***
FApr - Canola == 0    1.475324   0.095536  15.443    <0.01 ***
FC - Canola == 0      1.393121   0.095536  14.582    <0.01 ***
FP - Canola == 0      1.153577   0.095536  12.075    <0.01 ***
Soy - Canola == 0    -0.241482   0.095536  -2.528   0.3700
CD - CB == 0         -2.137127   0.095536 -22.370    <0.01 ***
CP - CB == 0         -0.017633   0.095536  -0.185   1.0000
FA - CB == 0          0.021867   0.095536   0.229   1.0000
FAlm - CB == 0        0.059196   0.095536   0.620   1.0000
FApr - CB == 0        0.148043   0.095536   1.550   0.9109
FC - CB == 0          0.065840   0.095536   0.689   0.9999
FP - CB == 0         -0.173704   0.095536  -1.818   0.7939
Soy - CB == 0        -1.568763   0.095536 -16.421    <0.01 ***
CP - CD == 0          2.119494   0.095536  22.185    <0.01 ***
FA - CD == 0          2.158994   0.095536  22.599    <0.01 ***
FAlm - CD == 0        2.196323   0.095536  22.990    <0.01 ***
FApr - CD == 0        2.285170   0.095536  23.920    <0.01 ***
FC - CD == 0          2.202967   0.095536  23.059    <0.01 ***
FP - CD == 0          1.963423   0.095536  20.552    <0.01 ***
Soy - CD == 0         0.568364   0.095536   5.949    <0.01 ***
FA - CP == 0          0.039500   0.095536   0.413   1.0000
FAlm - CP == 0        0.076829   0.095536   0.804   0.9994
FApr - CP == 0        0.165676   0.095536   1.734   0.8353
FC - CP == 0          0.083473   0.095536   0.874   0.9988
FP - CP == 0         -0.156071   0.095536  -1.634   0.8797
Soy - CP == 0        -1.551130   0.095536 -16.236    <0.01 ***
FAlm - FA == 0        0.037329   0.095536   0.391   1.0000
FApr - FA == 0        0.126176   0.095536   1.321   0.9679
FC - FA == 0          0.043973   0.095536   0.460   1.0000
FP - FA == 0         -0.195571   0.095536  -2.047   0.6603
Soy - FA == 0        -1.590630   0.095536 -16.650    <0.01 ***
FApr - FAlm == 0      0.088847   0.095536   0.930   0.9980
FC - FAlm == 0        0.006644   0.095536   0.070   1.0000
FP - FAlm == 0       -0.232900   0.095536  -2.438   0.4198
Soy - FAlm == 0      -1.627959   0.095536 -17.040    <0.01 ***
FC - FApr == 0       -0.082203   0.095536  -0.860   0.9990
FP - FApr == 0       -0.321747   0.095536  -3.368   0.0825 .
Soy - FApr == 0      -1.716806   0.095536 -17.970    <0.01 ***
FP - FC == 0         -0.239544   0.095536  -2.507   0.3813
Soy - FC == 0        -1.634603   0.095536 -17.110    <0.01 ***
Soy - FP == 0        -1.395059   0.095536 -14.603    <0.01 ***
---
Signif. codes:  0 ‘***’ 0.001 ‘**’ 0.01 ‘*’ 0.05 ‘.’ 0.1 ‘ ’ 1
(Adjusted p values reported -- single-step method)


Borage CA  Canola  CB  CD   CP   FA   FAlm   FApr   FC    FP    Soy
"c"    "d"    "b"  "d" "a"  "d"  "d"  "d"    "d"    "d"   "cd"   "b"
```

Brassicasterol

```
            Df Sum Sq Mean Sq F value   Pr(>F)    
Samples     11 29.789  2.7081   13.45 1.01e-07 ***
Residuals   24  4.832  0.2013                     
---
Signif. codes:  0 ‘***’ 0.001 ‘**’ 0.01 ‘*’ 0.05 ‘.’ 0.1 ‘ ’ 1


Simultaneous Tests for General Linear Hypotheses

Multiple Comparisons of Means: Tukey Contrasts


Linear Hypotheses:
                     Estimate Std. Error t value Pr(>|t|)    
CA - Borage == 0     -0.80421    0.36638  -2.195   0.5677    
Canola - Borage == 0  1.94612    0.36638   5.312    <0.01 ***
CB - Borage == 0     -0.60225    0.36638  -1.644   0.8757    
CD - Borage == 0      0.90614    0.36638   2.473   0.4009    
CP - Borage == 0     -0.24124    0.36638  -0.658   0.9999    
FA - Borage == 0      0.58877    0.36638   1.607   0.8904    
FAlm - Borage == 0    1.38137    0.36638   3.770   0.0353 *  
FApr - Borage == 0   -0.15315    0.36638  -0.418   1.0000    
FC - Borage == 0     -0.66847    0.36638  -1.825   0.7903    
FP - Borage == 0      1.61879    0.36638   4.418    <0.01 ** 
Soy - Borage == 0     0.97192    0.36638   2.653   0.3067    
Canola - CA == 0      2.75033    0.36638   7.507    <0.01 ***
CB - CA == 0          0.20196    0.36638   0.551   1.0000    
CD - CA == 0          1.71034    0.36638   4.668    <0.01 ** 
CP - CA == 0          0.56297    0.36638   1.537   0.9150    
FA - CA == 0          1.39298    0.36638   3.802   0.0328 *  
FAlm - CA == 0        2.18558    0.36638   5.965    <0.01 ***
FApr - CA == 0        0.65106    0.36638   1.777   0.8146    
FC - CA == 0          0.13574    0.36638   0.370   1.0000    
FP - CA == 0          2.42299    0.36638   6.613    <0.01 ***
Soy - CA == 0         1.77613    0.36638   4.848    <0.01 ** 
CB - Canola == 0     -2.54837    0.36638  -6.956    <0.01 ***
CD - Canola == 0     -1.03999    0.36638  -2.839   0.2248    
CP - Canola == 0     -2.18736    0.36638  -5.970    <0.01 ***
FA - Canola == 0     -1.35735    0.36638  -3.705   0.0406 *  
FAlm - Canola == 0   -0.56475    0.36638  -1.541   0.9135    
FApr - Canola == 0   -2.09928    0.36638  -5.730    <0.01 ***
FC - Canola == 0     -2.61459    0.36638  -7.136    <0.01 ***
FP - Canola == 0     -0.32734    0.36638  -0.893   0.9985    
Soy - Canola == 0    -0.97420    0.36638  -2.659   0.3031    
CD - CB == 0          1.50838    0.36638   4.117   0.0159 *  
CP - CB == 0          0.36101    0.36638   0.985   0.9967    
FA - CB == 0          1.19102    0.36638   3.251   0.1044    
FAlm - CB == 0        1.98362    0.36638   5.414    <0.01 ***
FApr - CB == 0        0.44909    0.36638   1.226   0.9809    
FC - CB == 0         -0.06622    0.36638  -0.181   1.0000    
FP - CB == 0          2.22103    0.36638   6.062    <0.01 ***
Soy - CB == 0         1.57417    0.36638   4.297   0.0102 *  
CP - CD == 0         -1.14738    0.36638  -3.132   0.1306    
FA - CD == 0         -0.31737    0.36638  -0.866   0.9989    
FAlm - CD == 0        0.47524    0.36638   1.297   0.9716    
```

```
FApr - CD == 0       -1.05929    0.36638  -2.891   0.2049
FC - CD == 0         -1.57461    0.36638  -4.298   0.0102 *
FP - CD == 0          0.71265    0.36638   1.945   0.7222
Soy - CD == 0         0.06579    0.36638   0.180   1.0000
FA - CP == 0          0.83001    0.36638   2.265   0.5241
FAlm - CP == 0        1.62261    0.36638   4.429    <0.01 **
FApr - CP == 0        0.08809    0.36638   0.240   1.0000
FC - CP == 0         -0.42723    0.36638  -1.166   0.9870
FP - CP == 0          1.86003    0.36638   5.077    <0.01 **
Soy - CP == 0         1.21317    0.36638   3.311   0.0926 .
FAlm - FA == 0        0.79260    0.36638   2.163   0.5872
FApr - FA == 0       -0.74192    0.36638  -2.025   0.6740
FC - FA == 0         -1.25724    0.36638  -3.432   0.0722 .
FP - FA == 0          1.03002    0.36638   2.811   0.2348
Soy - FA == 0         0.38315    0.36638   1.046   0.9945
FApr - FAlm == 0     -1.53453    0.36638  -4.188   0.0132 *
FC - FAlm == 0       -2.04984    0.36638  -5.595    <0.01 ***
FP - FAlm == 0        0.23741    0.36638   0.648   0.9999
Soy - FAlm == 0      -0.40945    0.36638  -1.118   0.9907
FC - FApr == 0       -0.51532    0.36638  -1.407   0.9509
FP - FApr == 0        1.77194    0.36638   4.836    <0.01 **
Soy - FApr == 0       1.12508    0.36638   3.071   0.1486
FP - FC == 0          2.28726    0.36638   6.243    <0.01 ***
Soy - FC == 0         1.64039    0.36638   4.477    <0.01 **
Soy - FP == 0        -0.64686    0.36638  -1.766   0.8204
---
Signif. codes:  0 ‘***’ 0.001 ‘**’ 0.01 ‘*’ 0.05 ‘.’ 0.1 ‘ ’ 1
(Adjusted p values reported -- single-step method)

Borage   CA  Canola  CB    CD     CP     FA    FAlm    FApr    FC     FP     Soy
"ac"    "a"   "e"   "ab"  "ce"   "ac"  "bcd"  "de"   "ac"   "ab"   "de"   "ce"
```

B-sitosterol

```
            Df Sum Sq Mean Sq F value Pr(>F)    
Samples     11 31.016  2.8196   203.6 <2e-16 ***
Residuals   24  0.332  0.0139                   
---
Signif. codes:  0 ‘***’ 0.001 ‘**’ 0.01 ‘*’ 0.05 ‘.’ 0.1 ‘ ’ 1

Simultaneous Tests for General Linear Hypotheses

Multiple Comparisons of Means: Tukey Contrasts


Linear Hypotheses:
                     Estimate Std. Error t value Pr(>|t|)
CA - Borage == 0     -0.08289    0.09609  -0.863   0.9990
Canola - Borage == 0 -0.94910    0.09609  -9.877    <0.01 ***
CB - Borage == 0     -3.36322    0.09609 -34.999    <0.01 ***
CD - Borage == 0     -0.76830    0.09609  -7.995    <0.01 ***
CP - Borage == 0     -1.63908    0.09609 -17.057    <0.01 ***
FA - Borage == 0     -0.09835    0.09609  -1.023   0.9954
FAlm - Borage == 0   -0.12141    0.09609  -1.263   0.9764
FApr - Borage == 0   -0.27744    0.09609  -2.887   0.2071
FC - Borage == 0     -0.32976    0.09609  -3.432   0.0722 .
FP - Borage == 0     -0.13968    0.09609  -1.454   0.9395
Soy - Borage == 0    -1.03738    0.09609 -10.796    <0.01 ***
Canola - CA == 0     -0.86621    0.09609  -9.014    <0.01 ***
```

```
CB - CA == 0          -3.28033    0.09609 -34.137    <0.01 ***
CD - CA == 0          -0.68541    0.09609  -7.133    <0.01 ***
CP - CA == 0          -1.55619    0.09609 -16.195    <0.01 ***
FA - CA == 0          -0.01546    0.09609  -0.161   1.0000    
FAlm - CA == 0        -0.03852    0.09609  -0.401   1.0000    
FApr - CA == 0        -0.19455    0.09609  -2.025   0.6740    
FC - CA == 0          -0.24687    0.09609  -2.569   0.3480    
FP - CA == 0          -0.05679    0.09609  -0.591   1.0000    
Soy - CA == 0         -0.95449    0.09609  -9.933    <0.01 ***
CB - Canola == 0      -2.41412    0.09609 -25.123    <0.01 ***
CD - Canola == 0       0.18080    0.09609   1.881   0.7591    
CP - Canola == 0      -0.68998    0.09609  -7.180    <0.01 ***
FA - Canola == 0       0.85075    0.09609   8.853    <0.01 ***
FAlm - Canola == 0     0.82769    0.09609   8.613    <0.01 ***
FApr - Canola == 0     0.67166    0.09609   6.990    <0.01 ***
FC - Canola == 0       0.61934    0.09609   6.445    <0.01 ***
FP - Canola == 0       0.80942    0.09609   8.423    <0.01 ***
Soy - Canola == 0     -0.08828    0.09609  -0.919   0.9982    
CD - CB == 0           2.59492    0.09609  27.004    <0.01 ***
CP - CB == 0           1.72414    0.09609  17.942    <0.01 ***
FA - CB == 0           3.26488    0.09609  33.976    <0.01 ***
FAlm - CB == 0         3.24181    0.09609  33.736    <0.01 ***
FApr - CB == 0         3.08578    0.09609  32.112    <0.01 ***
FC - CB == 0           3.03346    0.09609  31.568    <0.01 ***
FP - CB == 0           3.22355    0.09609  33.546    <0.01 ***
Soy - CB == 0          2.32584    0.09609  24.204    <0.01 ***
CP - CD == 0          -0.87078    0.09609  -9.062    <0.01 ***
FA - CD == 0           0.66996    0.09609   6.972    <0.01 ***
FAlm - CD == 0         0.64689    0.09609   6.732    <0.01 ***
FApr - CD == 0         0.49087    0.09609   5.108    <0.01 ** 
FC - CD == 0           0.43854    0.09609   4.564    <0.01 ** 
FP - CD == 0           0.62863    0.09609   6.542    <0.01 ***
Soy - CD == 0         -0.26908    0.09609  -2.800   0.2416    
FA - CP == 0           1.54074    0.09609  16.034    <0.01 ***
FAlm - CP == 0         1.51767    0.09609  15.794    <0.01 ***
FApr - CP == 0         1.36165    0.09609  14.170    <0.01 ***
FC - CP == 0           1.30932    0.09609  13.625    <0.01 ***
FP - CP == 0           1.49941    0.09609  15.604    <0.01 ***
Soy - CP == 0          0.60170    0.09609   6.262    <0.01 ***
FAlm - FA == 0        -0.02306    0.09609  -0.240   1.0000    
FApr - FA == 0        -0.17909    0.09609  -1.864   0.7687    
FC - FA == 0          -0.23141    0.09609  -2.408   0.4366    
FP - FA == 0          -0.04133    0.09609  -0.430   1.0000    
Soy - FA == 0         -0.93904    0.09609  -9.772    <0.01 ***
FApr - FAlm == 0      -0.15603    0.09609  -1.624   0.8835    
FC - FAlm == 0        -0.20835    0.09609  -2.168   0.5854    
FP - FAlm == 0        -0.01827    0.09609  -0.190   1.0000    
Soy - FAlm == 0       -0.91597    0.09609  -9.532    <0.01 ***
FC - FApr == 0        -0.05232    0.09609  -0.544   1.0000    
FP - FApr == 0         0.13776    0.09609   1.434   0.9444    
Soy - FApr == 0       -0.75994    0.09609  -7.908    <0.01 ***
FP - FC == 0           0.19008    0.09609   1.978   0.7019    
Soy - FC == 0         -0.70762    0.09609  -7.364    <0.01 ***
Soy - FP == 0         -0.89770    0.09609  -9.342    <0.01 ***
---
Signif. codes:  0 ‘***’ 0.001 ‘**’ 0.01 ‘*’ 0.05 ‘.’ 0.1 ‘ ’ 1
(Adjusted p values reported -- single-step method)

Borage     CA Canola     CB     CD     CP     FA   FAlm   FApr     FC     FP 
Soy 
   "d"    "d"    "c"    "a"    "c"    "b"    "d"    "d"    "d"    "d"    "d" 
"c" 
```

Campestanol

```
            Df Sum Sq Mean Sq F value Pr(>F)
Samples     11  6.786  0.6169   1.844  0.102
Residuals   24  8.028  0.3345


Simultaneous Tests for General Linear Hypotheses

Multiple Comparisons of Means: Tukey Contrasts

Linear Hypotheses:
                     Estimate Std. Error t value Pr(>|t|)
CA - Borage == 0     -0.32081    0.47222  -0.679   0.9999
Canola - Borage == 0  0.08691    0.47222   0.184   1.0000
CB - Borage == 0      0.40639    0.47222   0.861   0.9990
CD - Borage == 0     -0.62994    0.47222  -1.334   0.9655
CP - Borage == 0      0.34430    0.47222   0.729   0.9998
FA - Borage == 0     -0.24425    0.47222  -0.517   1.0000
FAlm - Borage == 0    0.48692    0.47222   1.031   0.9951
FApr - Borage == 0   -0.46586    0.47222  -0.987   0.9966
FC - Borage == 0     -0.02234    0.47222  -0.047   1.0000
FP - Borage == 0      0.15003    0.47222   0.318   1.0000
Soy - Borage == 0     0.99276    0.47222   2.102   0.6256
Canola - CA == 0      0.40772    0.47222   0.863   0.9989
CB - CA == 0          0.72720    0.47222   1.540   0.9140
CD - CA == 0         -0.30914    0.47222  -0.655   0.9999
CP - CA == 0          0.66511    0.47222   1.408   0.9506
FA - CA == 0          0.07656    0.47222   0.162   1.0000
FAlm - CA == 0        0.80773    0.47222   1.711   0.8466
FApr - CA == 0       -0.14505    0.47222  -0.307   1.0000
FC - CA == 0          0.29847    0.47222   0.632   0.9999
FP - CA == 0          0.47084    0.47222   0.997   0.9963
Soy - CA == 0         1.31357    0.47222   2.782   0.2476
CB - Canola == 0      0.31948    0.47222   0.677   0.9999
CD - Canola == 0     -0.71685    0.47222  -1.518   0.9210
CP - Canola == 0      0.25739    0.47222   0.545   1.0000
FA - Canola == 0     -0.33116    0.47222  -0.701   0.9998
FAlm - Canola == 0    0.40001    0.47222   0.847   0.9991
FApr - Canola == 0   -0.55277    0.47222  -1.171   0.9865
FC - Canola == 0     -0.10925    0.47222  -0.231   1.0000
FP - Canola == 0      0.06312    0.47222   0.134   1.0000
Soy - Canola == 0     0.90585    0.47222   1.918   0.7380
CD - CB == 0         -1.03634    0.47222  -2.195   0.5669
CP - CB == 0         -0.06209    0.47222  -0.131   1.0000
FA - CB == 0         -0.65064    0.47222  -1.378   0.9572
FAlm - CB == 0        0.08053    0.47222   0.171   1.0000
FApr - CB == 0       -0.87225    0.47222  -1.847   0.7781
FC - CB == 0         -0.42873    0.47222  -0.908   0.9983
FP - CB == 0         -0.25636    0.47222  -0.543   1.0000
Soy - CB == 0         0.58637    0.47222   1.242   0.9792
CP - CD == 0          0.97424    0.47222   2.063   0.6502
FA - CD == 0          0.38569    0.47222   0.817   0.9994
FAlm - CD == 0        1.11686    0.47222   2.365   0.4622
FApr - CD == 0        0.16408    0.47222   0.347   1.0000
FC - CD == 0          0.60761    0.47222   1.287   0.9732
FP - CD == 0          0.77997    0.47222   1.652   0.8727
```

```
Soy - CD == 0          1.62270    0.47222   3.436   0.0716 .
FA - CP == 0          -0.58855    0.47222  -1.246   0.9786
FAlm - CP == 0         0.14262    0.47222   0.302   1.0000
FApr - CP == 0        -0.81016    0.47222  -1.716   0.8438
FC - CP == 0          -0.36664    0.47222  -0.776   0.9996
FP - CP == 0          -0.19427    0.47222  -0.411   1.0000
Soy - CP == 0          0.64846    0.47222   1.373   0.9581
FAlm - FA == 0         0.73117    0.47222   1.548   0.9112
FApr - FA == 0        -0.22161    0.47222  -0.469   1.0000
FC - FA == 0           0.22191    0.47222   0.470   1.0000
FP - FA == 0           0.39428    0.47222   0.835   0.9992
Soy - FA == 0          1.23701    0.47222   2.620   0.3224
FApr - FAlm == 0      -0.95278    0.47222  -2.018   0.6779
FC - FAlm == 0        -0.50925    0.47222  -1.078   0.9929
FP - FAlm == 0        -0.33689    0.47222  -0.713   0.9998
Soy - FAlm == 0        0.50584    0.47222   1.071   0.9933
FC - FApr == 0         0.44352    0.47222   0.939   0.9978
FP - FApr == 0         0.61589    0.47222   1.304   0.9705
Soy - FApr == 0        1.45862    0.47222   3.089   0.1432
FP - FC == 0           0.17236    0.47222   0.365   1.0000
Soy - FC == 0          1.01509    0.47222   2.150   0.5957
Soy - FP == 0          0.84273    0.47222   1.785   0.8113
---
Signif. codes:  0 ‘***’ 0.001 ‘**’ 0.01 ‘*’ 0.05 ‘.’ 0.1 ‘ ’ 1
(Adjusted p values reported -- single-step method)

Borage  CA Canola  CB   CD   CP    FA   FAlm   FApr    FC    FP   Soy
"a"    "a"    "a"  "a"  "a"  "a"    "a"  "a"    "a"    "a"   "a"  "a"
```

Campesterol

```
            Df Sum Sq Mean Sq F value   Pr(>F)
Samples     11  6.616  0.6014   39.84 1.05e-12 ***
Residuals   24  0.362  0.0151
---
Signif. codes:  0 ‘***’ 0.001 ‘**’ 0.01 ‘*’ 0.05 ‘.’ 0.1 ‘ ’ 1


Simultaneous Tests for General Linear Hypotheses

Multiple Comparisons of Means: Tukey Contrasts

Linear Hypotheses:
                     Estimate Std. Error t value Pr(>|t|)
CA - Borage == 0     -1.30696    0.10032 -13.027    <0.01 ***
Canola - Borage == 0 -0.14214    0.10032  -1.417   0.9486
CB - Borage == 0     -1.22060    0.10032 -12.167    <0.01 ***
CD - Borage == 0     -0.49684    0.10032  -4.952    <0.01 **
CP - Borage == 0     -0.51635    0.10032  -5.147    <0.01 **
FA - Borage == 0     -1.04341    0.10032 -10.400    <0.01 ***
FAlm - Borage == 0   -0.95070    0.10032  -9.476    <0.01 ***
FApr - Borage == 0   -1.44958    0.10032 -14.449    <0.01 ***
FC - Borage == 0     -0.72732    0.10032  -7.250    <0.01 ***
FP - Borage == 0     -0.66908    0.10032  -6.669    <0.01 ***
```

```
Soy - Borage == 0    -0.68858    0.10032  -6.864    <0.01 ***
Canola - CA == 0      1.16483    0.10032  11.611    <0.01 ***
CB - CA == 0          0.08636    0.10032   0.861   0.9990
CD - CA == 0          0.81012    0.10032   8.075    <0.01 ***
CP - CA == 0          0.79061    0.10032   7.881    <0.01 ***
FA - CA == 0          0.26355    0.10032   2.627   0.3191
FAlm - CA == 0        0.35626    0.10032   3.551   0.0557 .
FApr - CA == 0       -0.14261    0.10032  -1.422   0.9474
FC - CA == 0          0.57965    0.10032   5.778    <0.01 ***
FP - CA == 0          0.63788    0.10032   6.358    <0.01 ***
Soy - CA == 0         0.61838    0.10032   6.164    <0.01 ***
CB - Canola == 0     -1.07847    0.10032 -10.750    <0.01 ***
CD - Canola == 0     -0.35470    0.10032  -3.536   0.0586 .
CP - Canola == 0     -0.37421    0.10032  -3.730   0.0383 *
FA - Canola == 0     -0.90127    0.10032  -8.984    <0.01 ***
FAlm - Canola == 0   -0.80857    0.10032  -8.059    <0.01 ***
FApr - Canola == 0   -1.30744    0.10032 -13.032    <0.01 ***
FC - Canola == 0     -0.58518    0.10032  -5.833    <0.01 ***
FP - Canola == 0     -0.52695    0.10032  -5.252    <0.01 **
Soy - Canola == 0    -0.54645    0.10032  -5.447    <0.01 ***
CD - CB == 0          0.72376    0.10032   7.214    <0.01 ***
CP - CB == 0          0.70425    0.10032   7.020    <0.01 ***
FA - CB == 0          0.17720    0.10032   1.766   0.8200
FAlm - CB == 0        0.26990    0.10032   2.690   0.2878
FApr - CB == 0       -0.22897    0.10032  -2.282   0.5129
FC - CB == 0          0.49329    0.10032   4.917    <0.01 **
FP - CB == 0          0.55152    0.10032   5.497    <0.01 ***
Soy - CB == 0         0.53202    0.10032   5.303    <0.01 ***
CP - CD == 0         -0.01951    0.10032  -0.194   1.0000
FA - CD == 0         -0.54657    0.10032  -5.448    <0.01 ***
FAlm - CD == 0       -0.45386    0.10032  -4.524    <0.01 **
FApr - CD == 0       -0.95274    0.10032  -9.497    <0.01 ***
FC - CD == 0         -0.23048    0.10032  -2.297   0.5049
FP - CD == 0         -0.17225    0.10032  -1.717   0.8439
Soy - CD == 0        -0.19174    0.10032  -1.911   0.7419
FA - CP == 0         -0.52706    0.10032  -5.254    <0.01 **
FAlm - CP == 0       -0.43435    0.10032  -4.329    <0.01 **
FApr - CP == 0       -0.93322    0.10032  -9.302    <0.01 ***
FC - CP == 0         -0.21097    0.10032  -2.103   0.6260
FP - CP == 0         -0.15273    0.10032  -1.522   0.9193
Soy - CP == 0        -0.17223    0.10032  -1.717   0.8441
FAlm - FA == 0        0.09271    0.10032   0.924   0.9980
FApr - FA == 0       -0.40617    0.10032  -4.049   0.0187 *
FC - FA == 0          0.31609    0.10032   3.151   0.1269
FP - FA == 0          0.37432    0.10032   3.731   0.0378 *
Soy - FA == 0         0.35483    0.10032   3.537   0.0578 .
FApr - FAlm == 0     -0.49887    0.10032  -4.973    <0.01 **
FC - FAlm == 0        0.22338    0.10032   2.227   0.5476
FP - FAlm == 0        0.28162    0.10032   2.807   0.2378
Soy - FAlm == 0       0.26212    0.10032   2.613   0.3251
FC - FApr == 0        0.72226    0.10032   7.199    <0.01 ***
FP - FApr == 0        0.78049    0.10032   7.780    <0.01 ***
Soy - FApr == 0       0.76099    0.10032   7.585    <0.01 ***
FP - FC == 0          0.05823    0.10032   0.580   1.0000
Soy - FC == 0         0.03874    0.10032   0.386   1.0000
Soy - FP == 0        -0.01950    0.10032  -0.194   1.0000
---
```

```
Signif. codes:  0 ‘***’ 0.001 ‘**’ 0.01 ‘*’ 0.05 ‘.’ 0.1 ‘ ’ 1
(Adjusted p values reported -- single-step method)

Borage   CA Canola  CB   CD    CP    FA   FAlm   FApr  FC    FP   Soy
 "g"   "ab" "fg"  "ab" "ef"   "e"  "bc"  "bd"   "a"  "cde"  "de""cde"
```

Cholesterol

```
            Df Sum Sq Mean Sq F value Pr(>F)
Samples     11  2.639  0.2399   1.585  0.167
Residuals   24  3.632  0.1513


Simultaneous Tests for General Linear Hypotheses

Multiple Comparisons of Means: Tukey Contrasts


Linear Hypotheses:
                       Estimate Std. Error t value Pr(>|t|)
CA - Borage == 0     -5.073e-02  3.176e-01  -0.160    1.000
Canola - Borage == 0 -8.318e-02  3.176e-01  -0.262    1.000
CB - Borage == 0     -2.405e-01  3.176e-01  -0.757    1.000
CD - Borage == 0      4.068e-01  3.176e-01   1.281    0.974
CP - Borage == 0     -1.646e-01  3.176e-01  -0.518    1.000
FA - Borage == 0     -2.405e-01  3.176e-01  -0.757    1.000
FAlm - Borage == 0    6.559e-01  3.176e-01   2.065    0.649
FApr - Borage == 0    1.032e-01  3.176e-01   0.325    1.000
FC - Borage == 0     -2.405e-01  3.176e-01  -0.757    1.000
FP - Borage == 0      7.390e-02  3.176e-01   0.233    1.000
Soy - Borage == 0    -2.405e-01  3.176e-01  -0.757    1.000
Canola - CA == 0     -3.245e-02  3.176e-01  -0.102    1.000
CB - CA == 0         -1.898e-01  3.176e-01  -0.597    1.000
CD - CA == 0          4.575e-01  3.176e-01   1.440    0.943
CP - CA == 0         -1.139e-01  3.176e-01  -0.359    1.000
FA - CA == 0         -1.898e-01  3.176e-01  -0.597    1.000
FAlm - CA == 0        7.066e-01  3.176e-01   2.225    0.549
FApr - CA == 0        1.539e-01  3.176e-01   0.484    1.000
FC - CA == 0         -1.898e-01  3.176e-01  -0.597    1.000
FP - CA == 0          1.246e-01  3.176e-01   0.392    1.000
Soy - CA == 0        -1.898e-01  3.176e-01  -0.597    1.000
CB - Canola == 0     -1.573e-01  3.176e-01  -0.495    1.000
CD - Canola == 0      4.899e-01  3.176e-01   1.542    0.913
CP - Canola == 0     -8.147e-02  3.176e-01  -0.256    1.000
FA - Canola == 0     -1.573e-01  3.176e-01  -0.495    1.000
FAlm - Canola == 0    7.390e-01  3.176e-01   2.327    0.486
FApr - Canola == 0    1.863e-01  3.176e-01   0.587    1.000
FC - Canola == 0     -1.573e-01  3.176e-01  -0.495    1.000
FP - Canola == 0      1.571e-01  3.176e-01   0.495    1.000
Soy - Canola == 0    -1.573e-01  3.176e-01  -0.495    1.000
CD - CB == 0          6.472e-01  3.176e-01   2.038    0.666
CP - CB == 0          7.584e-02  3.176e-01   0.239    1.000
FA - CB == 0         -2.776e-17  3.176e-01   0.000    1.000
FAlm - CB == 0        8.963e-01  3.176e-01   2.822    0.232
```

```
FApr - CB == 0         3.436e-01  3.176e-01   1.082    0.993
FC - CB == 0          -5.551e-17  3.176e-01   0.000    1.000
FP - CB == 0           3.144e-01  3.176e-01   0.990    0.997
Soy - CB == 0          2.220e-16  3.176e-01   0.000    1.000
CP - CD == 0          -5.714e-01  3.176e-01  -1.799    0.804
FA - CD == 0          -6.472e-01  3.176e-01  -2.038    0.667
FAlm - CD == 0         2.491e-01  3.176e-01   0.784    1.000
FApr - CD == 0        -3.036e-01  3.176e-01  -0.956    0.997
FC - CD == 0          -6.472e-01  3.176e-01  -2.038    0.666
FP - CD == 0          -3.329e-01  3.176e-01  -1.048    0.994
Soy - CD == 0         -6.472e-01  3.176e-01  -2.038    0.666
FA - CP == 0          -7.584e-02  3.176e-01  -0.239    1.000
FAlm - CP == 0         8.205e-01  3.176e-01   2.583    0.341
FApr - CP == 0         2.678e-01  3.176e-01   0.843    0.999
FC - CP == 0          -7.584e-02  3.176e-01  -0.239    1.000
FP - CP == 0           2.385e-01  3.176e-01   0.751    1.000
Soy - CP == 0         -7.584e-02  3.176e-01  -0.239    1.000
FAlm - FA == 0         8.963e-01  3.176e-01   2.822    0.232
FApr - FA == 0         3.436e-01  3.176e-01   1.082    0.993
FC - FA == 0          -2.776e-17  3.176e-01   0.000    1.000
FP - FA == 0           3.144e-01  3.176e-01   0.990    0.997
Soy - FA == 0          2.498e-16  3.176e-01   0.000    1.000
FApr - FAlm == 0      -5.527e-01  3.176e-01  -1.740    0.833
FC - FAlm == 0        -8.963e-01  3.176e-01  -2.822    0.233
FP - FAlm == 0        -5.820e-01  3.176e-01  -1.832    0.786
Soy - FAlm == 0       -8.963e-01  3.176e-01  -2.822    0.232
FC - FApr == 0        -3.436e-01  3.176e-01  -1.082    0.993
FP - FApr == 0        -2.925e-02  3.176e-01  -0.092    1.000
Soy - FApr == 0       -3.436e-01  3.176e-01  -1.082    0.993
FP - FC == 0           3.144e-01  3.176e-01   0.990    0.997
Soy - FC == 0          2.776e-16  3.176e-01   0.000    1.000
Soy - FP == 0         -3.144e-01  3.176e-01  -0.990    0.997
(Adjusted p values reported -- single-step method)

Borage    CA Canola   CB    CD     CP     FA   FAlm   FApr     FC     FP    Soy
"a"      "a"  "a"    "a"   "a"    "a"    "a"    "a"    "a"    "a"    "a"   "a"
```

Delta5-avenasterol

```
            Df Sum Sq Mean Sq F value   Pr(>F)
Samples     11  4.001  0.3637   21.33 9.32e-10 ***
Residuals   24  0.409  0.0171
---
Signif. codes:  0 ‘***’ 0.001 ‘**’ 0.01 ‘*’ 0.05 ‘.’ 0.1 ‘ ’ 1


Simultaneous Tests for General Linear Hypotheses

Multiple Comparisons of Means: Tukey Contrasts

Linear Hypotheses:
                     Estimate Std. Error t value Pr(>|t|)
CA - Borage == 0     -0.07002    0.10663  -0.657  0.99992
Canola - Borage == 0 -0.58736    0.10663  -5.509  < 0.001 ***
```

CB - Borage == 0	-0.09292	0.10663	-0.871	0.99885	
CD - Borage == 0	-0.67976	0.10663	-6.375	< 0.001	***
CP - Borage == 0	-0.54191	0.10663	-5.082	0.00172	**
FA - Borage == 0	0.07396	0.10663	0.694	0.99986	
FAlm - Borage == 0	-0.23924	0.10663	-2.244	0.53642	
FApr - Borage == 0	-0.01894	0.10663	-0.178	1.00000	
FC - Borage == 0	0.11661	0.10663	1.094	0.99209	
FP - Borage == 0	0.02867	0.10663	0.269	1.00000	
Soy - Borage == 0	-0.92838	0.10663	-8.707	< 0.001	***
Canola - CA == 0	-0.51734	0.10663	-4.852	0.00280	**
CB - CA == 0	-0.02291	0.10663	-0.215	1.00000	
CD - CA == 0	-0.60974	0.10663	-5.719	< 0.001	***
CP - CA == 0	-0.47189	0.10663	-4.426	0.00766	**
FA - CA == 0	0.14398	0.10663	1.350	0.96247	
FAlm - CA == 0	-0.16922	0.10663	-1.587	0.89779	
FApr - CA == 0	0.05108	0.10663	0.479	1.00000	
FC - CA == 0	0.18663	0.10663	1.750	0.82853	
FP - CA == 0	0.09869	0.10663	0.926	0.99804	
Soy - CA == 0	-0.85837	0.10663	-8.050	< 0.001	***
CB - Canola == 0	0.49444	0.10663	4.637	0.00462	**
CD - Canola == 0	-0.09240	0.10663	-0.867	0.99890	
CP - Canola == 0	0.04545	0.10663	0.426	1.00000	
FA - Canola == 0	0.66133	0.10663	6.202	< 0.001	***
FAlm - Canola == 0	0.34812	0.10663	3.265	0.10206	
FApr - Canola == 0	0.56842	0.10663	5.331	< 0.001	***
FC - Canola == 0	0.70397	0.10663	6.602	< 0.001	***
FP - Canola == 0	0.61603	0.10663	5.777	< 0.001	***
Soy - Canola == 0	-0.34102	0.10663	-3.198	0.11605	
CD - CB == 0	-0.58684	0.10663	-5.504	< 0.001	***
CP - CB == 0	-0.44898	0.10663	-4.211	0.01270	*
FA - CB == 0	0.16689	0.10663	1.565	0.90530	
FAlm - CB == 0	-0.14631	0.10663	-1.372	0.95827	
FApr - CB == 0	0.07398	0.10663	0.694	0.99986	
FC - CB == 0	0.20953	0.10663	1.965	0.70990	
FP - CB == 0	0.12159	0.10663	1.140	0.98904	
Soy - CB == 0	-0.83546	0.10663	-7.835	< 0.001	***
CP - CD == 0	0.13785	0.10663	1.293	0.97224	
FA - CD == 0	0.75372	0.10663	7.069	< 0.001	***
FAlm - CD == 0	0.44052	0.10663	4.131	0.01528	*
FApr - CD == 0	0.66082	0.10663	6.198	< 0.001	***
FC - CD == 0	0.79637	0.10663	7.469	< 0.001	***
FP - CD == 0	0.70843	0.10663	6.644	< 0.001	***
Soy - CD == 0	-0.24862	0.10663	-2.332	0.48300	
FA - CP == 0	0.61587	0.10663	5.776	< 0.001	***
FAlm - CP == 0	0.30267	0.10663	2.839	0.22547	
FApr - CP == 0	0.52297	0.10663	4.905	0.00246	**
FC - CP == 0	0.65852	0.10663	6.176	< 0.001	***
FP - CP == 0	0.57058	0.10663	5.351	< 0.001	***
Soy - CP == 0	-0.38648	0.10663	-3.625	0.04787	*
FAlm - FA == 0	-0.31320	0.10663	-2.937	0.18979	
FApr - FA == 0	-0.09291	0.10663	-0.871	0.99885	
FC - FA == 0	0.04264	0.10663	0.400	1.00000	
FP - FA == 0	-0.04530	0.10663	-0.425	1.00000	
Soy - FA == 0	-1.00235	0.10663	-9.401	< 0.001	***
FApr - FAlm == 0	0.22030	0.10663	2.066	0.64884	
FC - FAlm == 0	0.35585	0.10663	3.337	0.08713	.
FP - FAlm == 0	0.26791	0.10663	2.513	0.37846	

```
Soy - FAlm == 0       -0.68915    0.10663  -6.463  < 0.001 ***
FC - FApr == 0         0.13555    0.10663   1.271  0.97537
FP - FApr == 0         0.04761    0.10663   0.447  1.00000
Soy - FApr == 0       -0.90944    0.10663  -8.529  < 0.001 ***
FP - FC == 0          -0.08794    0.10663  -0.825  0.99928
Soy - FC == 0         -1.04499    0.10663  -9.801  < 0.001 ***
Soy - FP == 0         -0.95705    0.10663  -8.976  < 0.001 ***
---
Signif. codes:  0 ‘***’ 0.001 ‘**’ 0.01 ‘*’ 0.05 ‘.’ 0.1 ‘ ’ 1
(Adjusted p values reported -- single-step method)

Borage   CA Canola   CB   CD    CP    FA    FAlm    FApr    FC    FP  Soy
 "d"    "d"   "ac"  "d" "ab" "bc"  "d"   "cd"    "d"    "d"   "d" "a"
```

Desmosterol

```
            Df Sum Sq Mean Sq F value   Pr(>F)
Samples     11 22.379  2.0344   5.988 0.000126 ***
Residuals   24  8.155  0.3398
---
Signif. codes:  0 ‘***’ 0.001 ‘**’ 0.01 ‘*’ 0.05 ‘.’ 0.1 ‘ ’ 1


Simultaneous Tests for General Linear Hypotheses

Fit: aov(formula = Desmo ~ Samples, na.action = na.omit)

Linear Hypotheses:
                       Estimate Std. Error t value Pr(>|t|)
CA - Borage == 0      2.017e+00  4.759e-01   4.238   0.0121 *
Canola - Borage == 0  1.717e+00  4.759e-01   3.607   0.0505 .
CB - Borage == 0      1.768e+00  4.759e-01   3.715   0.0395 *
CD - Borage == 0      1.937e+00  4.759e-01   4.071   0.0184 *
CP - Borage == 0      6.131e-01  4.759e-01   1.288   0.9731
FA - Borage == 0      1.688e+00  4.759e-01   3.547   0.0569 .
FAlm - Borage == 0    1.948e+00  4.759e-01   4.094   0.0167 *
FApr - Borage == 0    1.155e+00  4.759e-01   2.426   0.4264
FC - Borage == 0      1.894e-02  4.759e-01   0.040   1.0000
FP - Borage == 0      1.691e+00  4.759e-01   3.553   0.0559 .
Soy - Borage == 0     2.731e-15  4.759e-01   0.000   1.0000
Canola - CA == 0     -3.003e-01  4.759e-01  -0.631   0.9999
CB - CA == 0         -2.489e-01  4.759e-01  -0.523   1.0000
CD - CA == 0         -7.965e-02  4.759e-01  -0.167   1.0000
CP - CA == 0         -1.404e+00  4.759e-01  -2.950   0.1855
FA - CA == 0         -3.288e-01  4.759e-01  -0.691   0.9999
FAlm - CA == 0       -6.864e-02  4.759e-01  -0.144   1.0000
FApr - CA == 0       -8.623e-01  4.759e-01  -1.812   0.7965
FC - CA == 0         -1.998e+00  4.759e-01  -4.198   0.0131 *
FP - CA == 0         -3.259e-01  4.759e-01  -0.685   0.9999
Soy - CA == 0        -2.017e+00  4.759e-01  -4.238   0.0116 *
CB - Canola == 0      5.134e-02  4.759e-01   0.108   1.0000
CD - Canola == 0      2.206e-01  4.759e-01   0.464   1.0000
CP - Canola == 0     -1.104e+00  4.759e-01  -2.319   0.4913
FA - Canola == 0     -2.850e-02  4.759e-01  -0.060   1.0000
```

```
FAlm - Canola == 0    2.316e-01  4.759e-01   0.487   1.0000
FApr - Canola == 0   -5.620e-01  4.759e-01  -1.181   0.9857
FC - Canola == 0     -1.698e+00  4.759e-01  -3.567   0.0546 .
FP - Canola == 0     -2.562e-02  4.759e-01  -0.054   1.0000
Soy - Canola == 0    -1.717e+00  4.759e-01  -3.607   0.0495 *
CD - CB == 0          1.693e-01  4.759e-01   0.356   1.0000
CP - CB == 0         -1.155e+00  4.759e-01  -2.427   0.4258
FA - CB == 0         -7.984e-02  4.759e-01  -0.168   1.0000
FAlm - CB == 0        1.803e-01  4.759e-01   0.379   1.0000
FApr - CB == 0       -6.134e-01  4.759e-01  -1.289   0.9727
FC - CB == 0         -1.749e+00  4.759e-01  -3.675   0.0431 *
FP - CB == 0         -7.696e-02  4.759e-01  -0.162   1.0000
Soy - CB == 0        -1.768e+00  4.759e-01  -3.715   0.0396 *
CP - CD == 0         -1.324e+00  4.759e-01  -2.782   0.2470
FA - CD == 0         -2.491e-01  4.759e-01  -0.523   1.0000
FAlm - CD == 0        1.101e-02  4.759e-01   0.023   1.0000
FApr - CD == 0       -7.827e-01  4.759e-01  -1.644   0.8755
FC - CD == 0         -1.918e+00  4.759e-01  -4.031   0.0195 *
FP - CD == 0         -2.463e-01  4.759e-01  -0.517   1.0000
Soy - CD == 0        -1.937e+00  4.759e-01  -4.071   0.0179 *
FA - CP == 0          1.075e+00  4.759e-01   2.259   0.5267
FAlm - CP == 0        1.335e+00  4.759e-01   2.806   0.2376
FApr - CP == 0        5.416e-01  4.759e-01   1.138   0.9892
FC - CP == 0         -5.941e-01  4.759e-01  -1.248   0.9784
FP - CP == 0          1.078e+00  4.759e-01   2.265   0.5245
Soy - CP == 0        -6.131e-01  4.759e-01  -1.288   0.9729
FAlm - FA == 0        2.601e-01  4.759e-01   0.547   1.0000
FApr - FA == 0       -5.335e-01  4.759e-01  -1.121   0.9904
FC - FA == 0         -1.669e+00  4.759e-01  -3.507   0.0616 .
FP - FA == 0          2.877e-03  4.759e-01   0.006   1.0000
Soy - FA == 0        -1.688e+00  4.759e-01  -3.547   0.0565 .
FApr - FAlm == 0     -7.937e-01  4.759e-01  -1.668   0.8657
FC - FAlm == 0       -1.929e+00  4.759e-01  -4.054   0.0183 *
FP - FAlm == 0       -2.573e-01  4.759e-01  -0.541   1.0000
Soy - FAlm == 0      -1.948e+00  4.759e-01  -4.094   0.0168 *
FC - FApr == 0       -1.136e+00  4.759e-01  -2.386   0.4499
FP - FApr == 0        5.364e-01  4.759e-01   1.127   0.9900
Soy - FApr == 0      -1.155e+00  4.759e-01  -2.426   0.4276
FP - FC == 0          1.672e+00  4.759e-01   3.513   0.0612 .
Soy - FC == 0        -1.894e-02  4.759e-01  -0.040   1.0000
Soy - FP == 0        -1.691e+00  4.759e-01  -3.553   0.0552 .
---
Signif. codes:  0 ‘***’ 0.001 ‘**’ 0.01 ‘*’ 0.05 ‘.’ 0.1 ‘ ’ 1
(Adjusted p values reported -- single-step method)

Borage   CA Canola  CB   CD   CP   FA  FAlm   FApr   FC   FP   Soy
"a"    "b"   "ab"  "b"  "b" "ab" "ab"  "b"   "ab"   "a"  "ab" "a"
```

Ergosterol

```
            Df Sum Sq Mean Sq F value   Pr(>F)
Samples     11 13.116  1.1924   12.52 2.01e-07 ***
Residuals   24  2.286  0.0952
---
```

```
Signif. codes:  0 ‘***’ 0.001 ‘**’ 0.01 ‘*’ 0.05 ‘.’ 0.1 ‘ ’ 1


Simultaneous Tests for General Linear Hypotheses

Multiple Comparisons of Means: Tukey Contrasts

Linear Hypotheses:
                     Estimate Std. Error t value Pr(>|t|)    
CA - Borage == 0     -0.18087    0.25197  -0.718  0.99981    
Canola - Borage == 0 -0.27011    0.25197  -1.072  0.99330    
CB - Borage == 0     -0.34297    0.25197  -1.361  0.96040    
CD - Borage == 0      1.27677    0.25197   5.067  0.00162 ** 
CP - Borage == 0     -1.00614    0.25197  -3.993  0.02106 *  
FA - Borage == 0     -1.08122    0.25197  -4.291  0.01079 *  
FAlm - Borage == 0   -0.44571    0.25197  -1.769  0.81903    
FApr - Borage == 0   -0.91263    0.25197  -3.622  0.04838 *  
FC - Borage == 0     -0.61804    0.25197  -2.453  0.41156    
FP - Borage == 0     -0.79019    0.25197  -3.136  0.13041    
Soy - Borage == 0    -0.59654    0.25197  -2.368  0.46129    
Canola - CA == 0     -0.08924    0.25197  -0.354  1.00000    
CB - CA == 0         -0.16209    0.25197  -0.643  0.99993    
CD - CA == 0          1.45765    0.25197   5.785  < 0.001 ***
CP - CA == 0         -0.82527    0.25197  -3.275  0.09931 .  
FA - CA == 0         -0.90035    0.25197  -3.573  0.05370 .  
FAlm - CA == 0       -0.26484    0.25197  -1.051  0.99426    
FApr - CA == 0       -0.73176    0.25197  -2.904  0.20099    
FC - CA == 0         -0.43716    0.25197  -1.735  0.83545    
FP - CA == 0         -0.60931    0.25197  -2.418  0.43156    
Soy - CA == 0        -0.41566    0.25197  -1.650  0.87310    
CB - Canola == 0     -0.07286    0.25197  -0.289  1.00000    
CD - Canola == 0      1.54688    0.25197   6.139  < 0.001 ***
CP - Canola == 0     -0.73603    0.25197  -2.921  0.19483    
FA - Canola == 0     -0.81111    0.25197  -3.219  0.11079    
FAlm - Canola == 0   -0.17560    0.25197  -0.697  0.99985    
FApr - Canola == 0   -0.64253    0.25197  -2.550  0.35832    
FC - Canola == 0     -0.34793    0.25197  -1.381  0.95655    
FP - Canola == 0     -0.52008    0.25197  -2.064  0.64997    
Soy - Canola == 0    -0.32643    0.25197  -1.296  0.97173    
CD - CB == 0          1.61974    0.25197   6.428  < 0.001 ***
CP - CB == 0         -0.66318    0.25197  -2.632  0.31589    
FA - CB == 0         -0.73826    0.25197  -2.930  0.19190    
FAlm - CB == 0       -0.10275    0.25197  -0.408  1.00000    
FApr - CB == 0       -0.56967    0.25197  -2.261  0.52612    
FC - CB == 0         -0.27507    0.25197  -1.092  0.99217    
FP - CB == 0         -0.44722    0.25197  -1.775  0.81611    
Soy - CB == 0        -0.25357    0.25197  -1.006  0.99600    
CP - CD == 0         -2.28292    0.25197  -9.060  < 0.001 ***
FA - CD == 0         -2.35800    0.25197  -9.358  < 0.001 ***
FAlm - CD == 0       -1.72249    0.25197  -6.836  < 0.001 ***
FApr - CD == 0       -2.18941    0.25197  -8.689  < 0.001 ***
FC - CD == 0         -1.89481    0.25197  -7.520  < 0.001 ***
FP - CD == 0         -2.06696    0.25197  -8.203  < 0.001 ***
Soy - CD == 0        -1.87331    0.25197  -7.435  < 0.001 ***
FA - CP == 0         -0.07508    0.25197  -0.298  1.00000    
FAlm - CP == 0        0.56043    0.25197   2.224  0.54937    
FApr - CP == 0        0.09351    0.25197   0.371  1.00000    
```

```
FC - CP == 0          0.38810    0.25197   1.540  0.91370
FP - CP == 0          0.21595    0.25197   0.857  0.99900
Soy - CP == 0         0.40960    0.25197   1.626  0.88289
FAlm - FA == 0        0.63551    0.25197   2.522  0.37286
FApr - FA == 0        0.16859    0.25197   0.669  0.99990
FC - FA == 0          0.46319    0.25197   1.838  0.78281
FP - FA == 0          0.29104    0.25197   1.155  0.98792
Soy - FA == 0         0.48469    0.25197   1.924  0.73472
FApr - FAlm == 0     -0.46692    0.25197  -1.853  0.77421
FC - FAlm == 0       -0.17233    0.25197  -0.684  0.99988
FP - FAlm == 0       -0.34448    0.25197  -1.367  0.95931
Soy - FAlm == 0      -0.15083    0.25197  -0.599  0.99997
FC - FApr == 0        0.29460    0.25197   1.169  0.98663
FP - FApr == 0        0.12245    0.25197   0.486  1.00000
Soy - FApr == 0       0.31610    0.25197   1.255  0.97768
FP - FC == 0         -0.17215    0.25197  -0.683  0.99988
Soy - FC == 0         0.02150    0.25197   0.085  1.00000
Soy - FP == 0         0.19365    0.25197   0.769  0.99964
---
Signif. codes:  0 '***' 0.001 '**' 0.01 '*' 0.05 '.' 0.1 ' ' 1
(Adjusted p values reported -- single-step method)

Borage CA Canola  CB  CD   CP  FA  FAlm  FApr  FC   FP  Soy
"b"   "ab" "ab"  "ab" "c"  "a" "a" "ab"  "a"   "ab" "ab""ab"
```

Sitostanol

```
            Df Sum Sq Mean Sq F value   Pr(>F)
Samples     11  9.607  0.8733   5.507 0.000239 ***
Residuals   24  3.806  0.1586
---
Signif. codes:  0 '***' 0.001 '**' 0.01 '*' 0.05 '.' 0.1 ' ' 1
Simultaneous Tests for General Linear Hypotheses

Fit: aov(formula = Sitostanol ~ Samples, na.action = na.omit)

Linear Hypotheses:
                         Estimate Std. Error t value Pr(>|t|)
CA - Borage == 0          0.02751    0.32517   0.085   1.0000
Canola - Borage == 0      1.11129    0.32517   3.418   0.0741 .
CB - Borage == 0          0.28402    0.32517   0.873   0.9988
CD - Borage == 0          1.91554    0.32517   5.891    <0.01 ***
CP - Borage == 0          0.97288    0.32517   2.992   0.1710
FA - Borage == 0          1.22627    0.32517   3.771   0.0349 *
FAlm - Borage == 0        0.71996    0.32517   2.214   0.5557
FApr - Borage == 0        0.78480    0.32517   2.414   0.4339
FC - Borage == 0          0.39320    0.32517   1.209   0.9828
FP - Borage == 0          0.82513    0.32517   2.538   0.3656
Soy - Borage == 0         0.75286    0.32517   2.315   0.4929
Canola - CA == 0          1.08378    0.32517   3.333   0.0884 .
CB - CA == 0              0.25651    0.32517   0.789   0.9995
CD - CA == 0              1.88803    0.32517   5.806    <0.01 ***
CP - CA == 0              0.94537    0.32517   2.907   0.2003
FA - CA == 0              1.19877    0.32517   3.687   0.0421 *
```

```
FAlm - CA == 0        0.69245    0.32517   2.130   0.6093
FApr - CA == 0        0.75729    0.32517   2.329   0.4846
FC - CA == 0          0.36569    0.32517   1.125   0.9902
FP - CA == 0          0.79763    0.32517   2.453   0.4110
Soy - CA == 0         0.72535    0.32517   2.231   0.5444
CB - Canola == 0     -0.82727    0.32517  -2.544   0.3610
CD - Canola == 0      0.80425    0.32517   2.473   0.3998
CP - Canola == 0     -0.13841    0.32517  -0.426   1.0000
FA - Canola == 0      0.11498    0.32517   0.354   1.0000
FAlm - Canola == 0   -0.39133    0.32517  -1.203   0.9834
FApr - Canola == 0   -0.32649    0.32517  -1.004   0.9961
FC - Canola == 0     -0.71809    0.32517  -2.208   0.5587
FP - Canola == 0     -0.28616    0.32517  -0.880   0.9987
Soy - Canola == 0    -0.35843    0.32517  -1.102   0.9916
CD - CB == 0          1.63152    0.32517   5.018    <0.01 **
CP - CB == 0          0.68886    0.32517   2.119   0.6151
FA - CB == 0          0.94226    0.32517   2.898   0.2037
FAlm - CB == 0        0.43594    0.32517   1.341   0.9641
FApr - CB == 0        0.50078    0.32517   1.540   0.9141
FC - CB == 0          0.10918    0.32517   0.336   1.0000
FP - CB == 0          0.54112    0.32517   1.664   0.8669
Soy - CB == 0         0.46884    0.32517   1.442   0.9424
CP - CD == 0         -0.94266    0.32517  -2.899   0.2029
FA - CD == 0         -0.68927    0.32517  -2.120   0.6145
FAlm - CD == 0       -1.19558    0.32517  -3.677   0.0426 *
FApr - CD == 0       -1.13074    0.32517  -3.477   0.0653 .
FC - CD == 0         -1.52234    0.32517  -4.682    <0.01 **
FP - CD == 0         -1.09041    0.32517  -3.353   0.0850 .
Soy - CD == 0        -1.16268    0.32517  -3.576   0.0532 .
FA - CP == 0          0.25339    0.32517   0.779   0.9996
FAlm - CP == 0       -0.25292    0.32517  -0.778   0.9996
FApr - CP == 0       -0.18808    0.32517  -0.578   1.0000
FC - CP == 0         -0.57968    0.32517  -1.783   0.8119
FP - CP == 0         -0.14775    0.32517  -0.454   1.0000
Soy - CP == 0        -0.22002    0.32517  -0.677   0.9999
FAlm - FA == 0       -0.50631    0.32517  -1.557   0.9084
FApr - FA == 0       -0.44147    0.32517  -1.358   0.9609
FC - FA == 0         -0.83307    0.32517  -2.562   0.3517
FP - FA == 0         -0.40114    0.32517  -1.234   0.9802
Soy - FA == 0        -0.47342    0.32517  -1.456   0.9387
FApr - FAlm == 0      0.06484    0.32517   0.199   1.0000
FC - FAlm == 0       -0.32676    0.32517  -1.005   0.9960
FP - FAlm == 0        0.10517    0.32517   0.323   1.0000
Soy - FAlm == 0       0.03290    0.32517   0.101   1.0000
FC - FApr == 0       -0.39160    0.32517  -1.204   0.9834
FP - FApr == 0        0.04033    0.32517   0.124   1.0000
Soy - FApr == 0      -0.03194    0.32517  -0.098   1.0000
FP - FC == 0          0.43193    0.32517   1.328   0.9665
Soy - FC == 0         0.35966    0.32517   1.106   0.9914
Soy - FP == 0        -0.07228    0.32517  -0.222   1.0000
---
Signif. codes:  0 ‘***’ 0.001 ‘**’ 0.01 ‘*’ 0.05 ‘.’ 0.1 ‘ ’ 1
(Adjusted p values reported -- single-step method)

Borage   CA Canola   CB    CD     CP    FA   FAlm   FApr     FC     FP    Soy
"a"     "a"   "ac" "ab"  "c"   "ac"  "bc"   "ab"   "ac"   "ab"   "ac"   "ac"
```

Stigmasterol

```
             Df Sum Sq Mean Sq F value   Pr(>F)    
Samples      11  9.186  0.8350   21.77 7.52e-10 ***
Residuals    24  0.921  0.0384                     
---
Signif. codes:  0 ‘***’ 0.001 ‘**’ 0.01 ‘*’ 0.05 ‘.’ 0.1 ‘ ’ 1


Simultaneous Tests for General Linear Hypotheses

Fit: aov(formula = Stigmasterol ~ Samples, na.action = na.omit)

Linear Hypotheses:
                      Estimate Std. Error t value Pr(>|t|)    
CA - Borage == 0      0.768583   0.159923   4.806    <0.01 ** 
Canola - Borage == 0 -0.371327   0.159923  -2.322   0.4888    
CB - Borage == 0      0.570414   0.159923   3.567   0.0548 .  
CD - Borage == 0      0.290692   0.159923   1.818   0.7939    
CP - Borage == 0      0.288881   0.159923   1.806   0.7998    
FA - Borage == 0      0.689940   0.159923   4.314   0.0101 *  
FAlm - Borage == 0    0.577650   0.159923   3.612   0.0491 *  
FApr - Borage == 0    0.618672   0.159923   3.869   0.0282 *  
FC - Borage == 0      0.493828   0.159923   3.088   0.1432    
FP - Borage == 0      0.826422   0.159923   5.168    <0.01 ** 
Soy - Borage == 0    -0.959374   0.159923  -5.999    <0.01 ***
Canola - CA == 0     -1.139910   0.159923  -7.128    <0.01 ***
CB - CA == 0         -0.198170   0.159923  -1.239   0.9795    
CD - CA == 0         -0.477891   0.159923  -2.988   0.1723    
CP - CA == 0         -0.479702   0.159923  -3.000   0.1682    
FA - CA == 0         -0.078643   0.159923  -0.492   1.0000    
FAlm - CA == 0       -0.190933   0.159923  -1.194   0.9844    
FApr - CA == 0       -0.149911   0.159923  -0.937   0.9978    
FC - CA == 0         -0.274755   0.159923  -1.718   0.8431    
FP - CA == 0          0.057839   0.159923   0.362   1.0000    
Soy - CA == 0        -1.727958   0.159923 -10.805    <0.01 ***
CB - Canola == 0      0.941741   0.159923   5.889    <0.01 ***
CD - Canola == 0      0.662019   0.159923   4.140   0.0151 *  
CP - Canola == 0      0.660208   0.159923   4.128   0.0154 *  
FA - Canola == 0      1.061267   0.159923   6.636    <0.01 ***
FAlm - Canola == 0    0.948977   0.159923   5.934    <0.01 ***
FApr - Canola == 0    0.989999   0.159923   6.190    <0.01 ***
FC - Canola == 0      0.865155   0.159923   5.410    <0.01 ***
FP - Canola == 0      1.197749   0.159923   7.490    <0.01 ***
Soy - Canola == 0    -0.588047   0.159923  -3.677   0.0428 *  
CD - CB == 0         -0.279721   0.159923  -1.749   0.8285    
CP - CB == 0         -0.281533   0.159923  -1.760   0.8231    
FA - CB == 0          0.119526   0.159923   0.747   0.9997    
FAlm - CB == 0        0.007236   0.159923   0.045   1.0000    
FApr - CB == 0        0.048259   0.159923   0.302   1.0000    
FC - CB == 0         -0.076586   0.159923  -0.479   1.0000    
FP - CB == 0          0.256009   0.159923   1.601   0.8926    
Soy - CB == 0        -1.529788   0.159923  -9.566    <0.01 ***
CP - CD == 0         -0.001811   0.159923  -0.011   1.0000    
FA - CD == 0          0.399248   0.159923   2.496   0.3872    
FAlm - CD == 0        0.286958   0.159923   1.794   0.8059    
FApr - CD == 0        0.327980   0.159923   2.051   0.6576    
```

```
FC - CD == 0          0.203136   0.159923   1.270   0.9756
FP - CD == 0          0.535730   0.159923   3.350   0.0857 .
Soy - CD == 0        -1.250067   0.159923  -7.817    <0.01 ***
FA - CP == 0          0.401059   0.159923   2.508   0.3801
FAlm - CP == 0        0.288769   0.159923   1.806   0.8002
FApr - CP == 0        0.329791   0.159923   2.062   0.6508
FC - CP == 0          0.204947   0.159923   1.282   0.9740
FP - CP == 0          0.537541   0.159923   3.361   0.0829 .
Soy - CP == 0        -1.248255   0.159923  -7.805    <0.01 ***
FAlm - FA == 0       -0.112290   0.159923  -0.702   0.9998
FApr - FA == 0       -0.071268   0.159923  -0.446   1.0000
FC - FA == 0         -0.196112   0.159923  -1.226   0.9811
FP - FA == 0          0.136482   0.159923   0.853   0.9990
Soy - FA == 0        -1.649314   0.159923 -10.313    <0.01 ***
FApr - FAlm == 0      0.041022   0.159923   0.257   1.0000
FC - FAlm == 0       -0.083822   0.159923  -0.524   1.0000
FP - FAlm == 0        0.248772   0.159923   1.556   0.9088
Soy - FAlm == 0      -1.537024   0.159923  -9.611    <0.01 ***
FC - FApr == 0       -0.124844   0.159923  -0.781   0.9996
FP - FApr == 0        0.207750   0.159923   1.299   0.9715
Soy - FApr == 0      -1.578047   0.159923  -9.868    <0.01 ***
FP - FC == 0          0.332594   0.159923   2.080   0.6404
Soy - FC == 0        -1.453202   0.159923  -9.087    <0.01 ***
Soy - FP == 0        -1.785797   0.159923 -11.167    <0.01 ***
---
Signif. codes:  0 ‘***’ 0.001 ‘**’ 0.01 ‘*’ 0.05 ‘.’ 0.1 ‘ ’ 1
(Adjusted p values reported -- single-step method)

Borage   CA Canola   CB   CD     CP    FA   FAlm   FApr   FC   FP   Soy
"bc"    "d"  "b"   "cd" "cd"   "cd"   "d"  "d"    "d"   "cd" "d"    "a"
```
